# E-Cigarette Use Among University Students: A Structured Literature Review of Health Risks, Behavioral and Social Determinants, and Nursing Implications

**DOI:** 10.3390/healthcare13172150

**Published:** 2025-08-28

**Authors:** Luis-Rodrigo Rocha-Ávila, María-Ángeles Núñez-Baila, José Rafael González-López

**Affiliations:** 1 Nursing Department, Faculty of Nursing, Physiotherapy and Podiatry, Universidad de Sevilla, 41009 Seville, Spain; 2 Instituto de Biomedicina de Sevilla, IBiS/Hospital Universitario Virgen del Rocío/CSIC/Universidad de Sevilla, 41013 Seville, Spain

**Keywords:** e-cigarettes, university students, health risks, social determinants of health, vaping, electronic nicotine delivery system, nursing education, review

## Abstract

**Background/Objectives**: E-cigarette use has increased substantially among university students in recent years, coinciding with a broader shift in nicotine consumption patterns globally. Despite initial perceptions of e-cigarettes as harm-reduction tools, growing evidence indicates significant health risks, misinformation, and limited awareness—especially within higher education environments. This structured literature review aims to synthesize peer-reviewed evidence on the health impacts, behavioral determinants, and the role of nursing in addressing e-cigarette use among university students. **Methods**: A literature search was conducted across five databases (PubMed, CINAHL, Scopus, Embase, Dialnet) between February and March 2025. Eligible studies were published between January 2020 and January 2025 in English or Spanish. A total of 43 studies were included. Data were synthesized narratively, and methodological quality was assessed using Joanna Briggs Institute checklists and The Scale for the Assessment of Narrative Reviews Articles. **Results**: E-cigarette use among university students showed wide variability in prevalence, with higher rates among males, students in non-health disciplines, and users of disposable devices. Key behavioral and social determinants included peer influence, curiosity, stress management, and social media exposure. Despite documented health risks—such as nicotine dependence, respiratory and cardiovascular impairment, and mental health concerns—misconceptions about safety and cessation efficacy were common, even among health science students. Nursing-led interventions hold great potential for prevention but remain underdeveloped within university settings. **Conclusions**: The findings underscore the urgent need for evidence-based prevention strategies—particularly those led by nurses—to reduce e-cigarette use, bridge knowledge gaps, and mitigate associated health risks in higher education. Future efforts should prioritize institutional policy reinforcement, improved health communication, and the integration of vaping-related education into nursing curricula and public health campaigns targeting emerging adults.

## 1. Introduction

Tobacco use remains one of the leading preventable causes of death worldwide, responsible for over 8 million deaths annually, including more than 1.3 million among non-smokers exposed to secondhand smoke [1]. The associated burden encompasses a wide range of cardiovascular and respiratory diseases, as well as more than 20 distinct types and subtypes of cancer [1]. In response to this public health crisis, electronic cigarettes (e-cigarettes) emerged in the early 2000s as a harm-reduction alternative to conventional tobacco products [2]. Consequently, the global landscape of nicotine consumption has undergone a profound transformation in recent decades, particularly among young individuals with no previous history of tobacco [2,3,4,5].

Globally, e-cigarette use has increased markedly over the past decade, particularly among adolescents and young adults. Between 2010 and 2014, the prevalence of e-cigarette use among adolescents aged 13–18 in Central and Eastern Europe rose by 24.4%, with 43.7% of university students in the region reporting ever-use [6]. In the European Union, approximately 48.5 million people have tried an e-cigarette at least once, and an estimated 7.5 million are current users [7]. In England, the prevalence of long-term (>6 months) vaping among adults increased from 1.3% in 2013 to 10.0% in 2023, with the most pronounced growth observed since 2021, coinciding with the rise in disposable e-cigarettes [8]. This upward trend was evident not only among current and former smokers but also among young adults with no history of regular smoking [8]. Comparable patterns of increased e-cigarette uptake have been reported in the United States, Canada, and Great Britain, with the phenomenon particularly well-documented in adolescent populations [9]. These prevalence trends illustrate the rapid uptake of e-cigarettes across diverse age groups and regions, including among individuals with no history of regular smoking. This widespread and accelerating use—particularly among young adults—underscores the urgency of examining not only patterns of consumption but also the associated health risks.

Although initially perceived as less harmful, e-cigarettes—including disposable versions—are not free of health risks [10,11]. Beyond nicotine, which remains the primary addictive component in most formulations, e-cigarette aerosols contain several potentially toxic and carcinogenic substances, including acrolein, formaldehyde, and polycyclic aromatic hydrocarbons [12]. Additionally, flavoring compounds such as diacetyl—associated with bronchiolitis obliterans—and solvents like propylene glycol and vegetable glycerin have been linked to respiratory irritation and increased mucin secretion [12,13,14,15,16,17]. Headaches, airway inflammation, and cellular toxicity have also been reported, underscoring the complex and underregulated nature of these products [12,13,14,15,16]. Emerging evidence highlights that, even in the absence of nicotine, repeated exposure to these chemicals may compromise pulmonary and systemic health.

Moreover, there is evidence that has detected urinary metabolites of several harmful compounds—such as acrylonitrile, acrolein, and propylene oxide—among e-cigarette users, confirming systemic exposure to toxicants other than nicotine [10]. These substances have been implicated in the disruption of pulmonary homeostasis, vascular integrity, and immune function, even in non-smokers exposed to secondhand aerosols [10]. In more severe cases, e-cigarette or vaping product use-associated lung injury (EVALI) has emerged as a clinical syndrome characterized by acute respiratory distress, severe pneumonia, and, in some instances, fatal outcomes [18]. Although its pathogenesis is multifactorial, evidence suggests that the combined inhalation of tetrahydrocannabinol (THC), flavoring agents, and other aerosolized chemicals may contribute synergistically to the development of this condition [19]. Notably, EVALI remains a diagnosis of exclusion, requiring the systematic ruling out of infectious, autoimmune, and other pulmonary causes before attribution to vaping-related exposure [20,21].

E-cigarettes are battery-powered devices that heat a liquid—commonly referred to as e-liquid or vape juice—to produce an inhalable aerosol [22]. Technological innovation has led to a broad spectrum of device types, which can be classified by generation and by the chemical composition of the e-liquid they use [23]. Four main technological generations are commonly recognized [24]: (1) first-generation “cigalikes”, which mimic traditional cigarettes in appearance and utilize disposable cartridges; (2) second-generation “vape pens”, featuring rechargeable batteries and refillable tanks; (3) third-generation “mods”, which are larger, customizable devices offering adjustable voltage and wattage; and (4) fourth-generation “pod systems”, which are compact, user-friendly devices that often use nicotine salts to deliver higher concentrations of nicotine with smoother inhalation.

In parallel, e-cigarettes can also be categorized based on the type of substances they aerosolize. E-liquids may contain nicotine (electronic nicotine delivery systems, ENDS), be nicotine-free (electronic non-nicotine delivery systems ENNDS), or include psychoactive compounds such as THC or cannabidiol (CBD) [25]. Many formulations also incorporate flavoring agents—ranging from sweet and fruity to mentholated and novelty flavors—which have been shown to increase product appeal, particularly among adolescents and young adults [26]. This combination of appealing flavors, discreet design, and targeted marketing has contributed to widespread misperceptions of e-cigarettes as harmless, despite growing evidence linking their use to a range of adverse health outcomes and to an increased likelihood of initiating or resuming conventional cigarette smoking [26].

University students constitute a population of particular concern due to their developmental stage and contextual vulnerabilities. Most fall within the period of emerging adulthood—typically defined as spanning ages 18 to 29—a life phase marked by transitions, identity exploration, increasing autonomy and economic and residential instability [27,28]. Concretely, experiencing a moderate number of role transitions (e.g., changes in romantic relationships or employment), compounded by reduced parental supervision and the growing social normalization of vaping, has been associated with increased likelihood of e-cigarette use. This suggests that experimentation may emerge either as a response to major life events or as a way to navigate a period characterized by ambiguity and exploration [29].

Although many universities have adopted tobacco- or smoke-free campus policies, recent evidence indicates that their implementation often lacks comprehensive strategies—such as effective communication, enforcement mechanisms, or access to cessation services. As a result, these environments may still foster experimentation and fail to adequately address the rising use of e-cigarettes, particularly in the absence of structured health education tailored to vaping behaviors [30]. Recent findings also indicate that many students actively circumvent institutional restrictions through behaviors such as stealth vaping—the discreet use of e-cigarettes in prohibited settings—by using small devices and low-visibility aerosols in locations such as bathrooms, libraries, classrooms, and parking garages [31].

The use of e-cigarettes among university students has increased notably in recent years, and they continue to be perceived as less harmful than conventional tobacco products—a misconception amplified by the smoother delivery of nicotine salts in pod-based systems, which may increase the potential for dependence [32]. Although research on this population is expanding, current evidence remains fragmented, particularly regarding usage patterns, motivations, and long-term outcomes [26]. Moreover, the role of healthcare professionals—especially nurses—has been largely overlooked, despite the relevance of university campuses as strategic settings for prevention, education, and tailored intervention programs [33,34]. While attention has been devoted to prevention efforts during childhood and adolescence, less focus has been placed on the university stage, where individuals, now legally adults, may have greater autonomy and easier access to e-cigarettes and other nicotine products [35].

Despite the growing body of literature on e-cigarette use, no review has comprehensively synthesized evidence on this behavior among university students while explicitly addressing the role of nursing in prevention and health promotion. A targeted search of PROSPERO conducted on 13 August 2025 identified no registered review with this scope. Other reviews that might appear to overlap at first glance [36,37] differ substantially once their objectives are considered: the former is dedicated to an exhaustive estimation of prevalence within a single regional and disciplinary context (medical students in Saudi Arabia), whereas the latter is a systematic review and meta-analysis assessing the efficacy of school-based preventive interventions targeting adolescents. Neither encompasses the broader university context or integrates evidence on usage patterns, health risks, behavioral and social determinants, and the role of nursing. In contrast, the present review synthesizes findings from multiple disciplines and countries, integrating epidemiological, behavioral, and social perspectives, and uniquely considers the implications for prevention and health promotion strategies through a nursing lens. This broader, interdisciplinary perspective addresses a critical gap and supports the development of targeted, evidence-based interventions to reduce vaping-related harm in higher education settings.

Drawing on a diverse body of evidence—including cross-sectional, longitudinal, mixed-methods studies, and literature reviews—this review examines e-cigarette use among university students, with particular emphasis on usage patterns, health risks and behavioral and social determinants. It also explores the implications for prevention and health promotion strategies, with a specific focus on the role of nursing. By situating vaping within the developmental context of emerging adulthood, this review aims to inform evidence-based public health responses and support targeted interventions that reduce vaping-related harm among university students in academic environments.

## 2. Materials and Methods

### 2.1. Design

This study is a structured literature review aiming to synthesize peer-reviewed evidence on the health impacts, behavioral and social determinants, and the role of nursing in addressing e-cigarette use among university students. Although systematic reviews offer a higher degree of methodological standardization, they are typically restricted to narrowly defined research questions and homogenous study designs, often excluding qualitative or mixed-methods evidence. In contrast, the structure approach adopted in this review allowed for the integration of a wide range of study types—including cross-sectional, longitudinal, qualitative, mixed-methods, and both systematic and non-systematic reviews—thereby enabling a more comprehensive synthesis of the complex and multifaceted evidence available. While a scoping review could also encompass diverse study designs, this methodology requires a pre-established protocol, ideally registered in a publicly accessible repository, which was not developed for this work. For this reason, and to ensure methodological rigor while retaining flexibility, a structured narrative format was selected. This review followed a structured approach to data retrieval, selection, analysis, and synthesis to ensure transparency and reproducibility. This review followed a structured approach to data retrieval, selection, analysis, and synthesis to ensure transparency and reproducibility.

### 2.2. Search Strategy

The literature search was conducted between February and March 2025 across five databases: PubMed, CINAHL, Scopus, Embase, and Dialnet. The following search strategies were used in both English and Spanish:

- (“Vaping” OR “Electronic Nicotine Delivery Systems” OR “e-cig*” OR “Electronic Cigarette”) AND (“Student*” OR “University Students”) AND (“Prevalence” OR “Epidemiology” OR “Incidence”)
- (“Vaping” OR “Electronic Nicotine Delivery Systems” OR “e-cig*” OR “Electronic Cigarette”) AND (“Student*” OR “University Students”) AND (“Nurs*” OR “Primary Care Nursing”)
- (“Vaping” OR “Electronic Nicotine Delivery Systems” OR “e-cig*” OR “Electronic Cigarette”) AND (“Student*” OR “University Students”) AND (“Health Promotion” OR “Student Health”)
- (“Vaping” OR “Electronic Nicotine Delivery Systems” OR “e-cig*” OR “Electronic Cigarette”) AND (“Student*” OR “University Students”) AND (“Tobacco Use Disorder” OR “Nicotine” OR “Substance-Related Disorders”) AND (“Clinical Pathology” OR “Signs and Symptoms”)

The results from the different search strategies were combined, and duplicates were removed using Zotero reference management software version 7.0.8 (Corporation for Digital Scholarship, Fairfax, VA, USA). The review was conducted following the Scale for the Assessment of Narrative Review Articles (SANRA) criteria for narrative reviews [38]. The structured approach and adherence to SANRA criteria ensured a transparent and methodologically sound synthesis of the available evidence.

### 2.3. Eligibility Criteria

Inclusion criteria:

- Articles published between January 2020 and January 2025.
- Written in English or Spanish.
- Focused on the harmful effects of e-cigarettes on the health of university students.
- No restrictions were placed on sex, age, or socioeconomic status within the student population.

Studies for which the full text was not accessible were excluded. In addition, other types of publications were not considered, including commentaries, clinical guidelines, dissertations, conference abstracts, editorial letters, PhD theses, books, book chapters, and reports.

### 2.4. Review Focus and Guiding Question (PICO Framework)

Although this review follows a narrative synthesis approach, the research question was structured using the PICO framework to enhance clarity and guide the literature search. This framework helped refine the scope of the review and identify relevant studies across a range of methodologies. However, it was not used to perform a meta-analysis or quantitative synthesis, due to the heterogeneity of study designs and outcomes.

- Population (P): University students (irrespective of biological sex, age, or discipline).
- Intervention/Exposure (I): Use of electronic cigarettes (vaping).
- Comparison (C): Not applicable or implicit (e.g., non-users or conventional smokers).
- Outcome (O): Health effects (e.g., respiratory, cardiovascular, and oral), usage patterns, beliefs, and the role of nursing in prevention and education.

This structure helped refine the scope of the review and optimize the identification of relevant literature.

### 2.5. Study Selection and Data Extraction

The first and third authors independently screened the titles and abstracts retrieved from the databases to identify studies that met the inclusion criteria. This initial screening was conducted manually, using a structured Excel spreadsheet, at the title/abstract/keywords level to exclude records that were clearly irrelevant, such as those not involving university students, not addressing e-cigarettes, or not focusing on health impacts, behavioral or social determinants, or nursing-related aspects. The process was followed by a full-text review of potentially eligible studies, with any doubts or discrepancies resolved through discussion with the second and third authors. Data extraction from full-text studies was conducted manually using a standardized Excel form developed for this review, which included predefined fields for study characteristics (author, year, country, design), population details, objectives, main findings, and information relevant to health impacts, behavioral and social determinants, and nursing-related aspects. The extracted data were then synthesized narratively and presented in a summary table in the 3. Results section.

### 2.6. Quality Appraisal

The methodological quality of the included studies was assessed using the design-specific checklists from the Joanna Briggs Institute (JBI), University of Adelaide, Adelaide, Australia and the SANRA tool for narrative reviews (Supplementary Material S1). The second author conducted the appraisal independently, and any discrepancies were resolved by consensus.

In line with the objectives of a structured literature review, no studies were excluded based on methodological quality alone. The appraisal served to contextualize the rigor of the included evidence and to inform the interpretation of findings. While no exclusion threshold was applied, numerical scores were assigned to facilitate quality classification. Studies meeting 100% of the checklist criteria were classified as “Excellent,” whereas those meeting between 70% and 99% were considered “Moderate to high quality.” Only one study received a score below this range (62.5%) and was rated as “Moderate quality”.

Among the 43 studies evaluated, most demonstrated a high methodological quality. Specifically, 22 studies (51.2%) met 100% of the assessed items, indicating full compliance with the quality appraisal tools. A total of 18 studies (41.9%) met between 75% and 99% of the checklist items, corresponding to a rating of Moderate to High Quality. This category includes studies that demonstrated acceptable methodological rigor but had at least one unmet criterion. Within this group, mixed-methods studies showed greater variability, with scores ranging from 6/8 (75%) and 7/10 (70%) for the respective quantitative and qualitative components, to 8/8 (100%) and 8/10 (80%) in the best-rated cases. These partial scores reflect specific methodological limitations, such as a lack of multivariable adjustment, an absence of reflexivity, or an insufficient validation of data collection instruments.

Among the four narrative reviews assessed using the SANDRA tool, scores ranged from 11/12 (87.5%) to 12/12 (100%), reflecting good adherence to standards of synthesis, transparency, and reasoning, though not all addressed aspects such as reproducibility or researcher reflexivity.

Only one study obtained a score of 5/8 (62.5%), classified as “Moderate” due to the absence of confounding control and a lack of validation of the measurement tools. A comprehensive summary of the methodological appraisal results for all included studies is available in Supplementary Material S2.

## 3. Results

### 3.1. Search Outcomes

**Figure 1:**
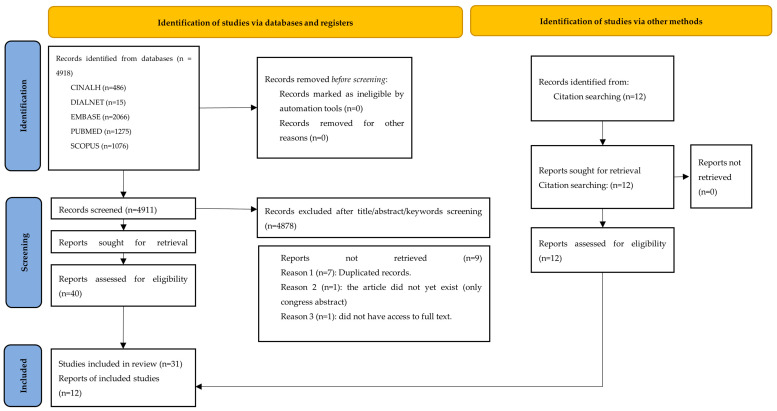
Article Selection Process.

An exhaustive search across five databases yielded a total of 4918 records. A large number of studies were excluded during the title/abstract/keywords screening stage, as illustrated in the PRISMA flow diagram (Figure 1). After applying the predefined inclusion and exclusion criteria and removing duplicates, 43 articles were retained for inclusion. Of these, 31 were identified through the initial database search and 12 through citation tracking. These studies, conducted in diverse regions using a variety of designs, are summarized in Table 1, and the article selection process is detailed in the PRISMA-style flow diagram (Figure 1).

### 3.2. Study Characteristics

The included studies were primarily original quantitative investigations, the majority of which followed a cross-sectional design (*n* = 34). Additionally, the review comprised one quasi-experimental study and three longitudinal or cohort studies. Three studies integrated both quantitative and qualitative approaches. While qualitative components were present, they were always embedded within mixed-methods frameworks rather than standing alone. Lastly, the review also incorporated four narrative reviews and one systematic review.

The studies included in this review were conducted across a wide range of countries, with the majority originating from the United States (*n* = 19). Additional studies were identified in Italy (*n* = 1), Saudi Arabia (*n* = 2), and Thailand (*n* = 2). Single studies were conducted in Spain and Portugal (jointly), China, New Zealand, Egypt, France, Jordan, Slovakia, Qatar, Chile, Pakistan, Germany, the Philippines, and the United Kingdom (specifically in Birmingham), reflecting a globally diverse yet predominantly Western-oriented research landscape. Table 1 outlines the characteristics of each study.

### 3.3. Thematic Synthesis of Results

In alignment with the aims of this structured literature review—to synthesize peer-reviewed evidence on the health impacts, behavioral determinants, and the role of nursing in addressing e-cigarette use among university students—the results were thematically organized into five interrelated domains. The synthesis begins with patterns and prevalence of use, providing a contextual foundation, followed by behavioral and social determinants as explanatory factors, and students’ knowledge and beliefs as perceptual mediators. These themes are subsequently linked to documented health outcomes and, lastly, to implications for healthcare professionals, with a particular emphasis on nursing. This thematic structure remains fully aligned with the scope and intent of the review, while enhancing analytical clarity by tracing a logical continuum from the epidemiological context to preventive action.

**Table 1:** Study Characteristics.

	Year	Study Location	Authorship	Study Desing	Study Objective	Main Findings/ Key Statistical Results
1	2020	United States	Dobbs et al. [39]	Mixed Methods Study	To evaluate the influences and sources through which young adults access information about vaping and how such information shapes their decisions.	In a sample of college students (*n* = 522), most information about e-cigarettes was obtained from peers (87.6%), while educational institutions were the least reported source (9.6%). Significant associations emerged between source of information and type of content: positive consequences were more frequently heard through advertising (χ^2^(1) = 9.58, *p* < 0.01), education (χ^2^(1) = 11.64, *p* < 0.01), and social sources (χ^2^(1) = 25.58, *p* < 0.001); negative consequences through education (χ^2^(1) = 17.91, *p* < 0.001) and media (χ^2^(1) = 8.31, *p* < 0.05); and cost/convenience through advertising (χ^2^(1) = 12.16, *p* < 0.001) and education (χ^2^(1) = 6.61, *p* < 0.05). Vaping status was significantly related to hearing about the negative consequences of e-cigarette use (χ^2^(1) = 15.65, *p* < 0.001) and to perceiving e-cigarettes as cessation aids (χ^2^(1) = 11.37, *p* < 0.01). No significant associations were found for hearing about vaping culture.
2	2020	Spain and Portugal	Fernández-García et al. [40]	Cross-Sectional Study	To assess tobacco use among undergraduate nursing students in Spanish universities and one Portuguese university.	A total of 1469 nursing students participated (79.8% response rate), Mean age 21.9 years, 80% female. Overall tobacco use prevalence (including e-cigarettes) was 20% (294/1469; 95% CI: 18–22.2), with 18.9% smoking cigarettes, 0.4% using e-cigarettes, and 0.7% dual use. Only two e-cigarette users reported recently quitting tobacco. Prevalence differed significantly by gender (24.4% men vs. 19% women; *p* < 0.05), previous studies (27.1% non–high school vs. 16.4% high school; *p* < 0.001), and university (e.g., León campus 43.5% vs. 19.7%; *p* < 0.001). Incidence of smoking initiation during university was 3.6%. Smokers reported low nicotine dependence (Fagerström M = 2.8 ± 2.0) and moderate motivation to quit (Richmond M = 4.9 ± 3.0). Living with smokers was more common among smokers (52.2% vs. 38.8%; *p* < 0.001). Male smokers were less likely to agree with the harms of secondhand smoke (OR = 2.4, 95% CI: 1.5–3.7), with particularly low agreement among male smokers overall (OR = 6.7, 95% CI: 4.3–10.5, *p* < 0.001).
3	2020	-	Huey et al. [41]	Narrative Review	To analyze EVALI and emphasize the role of nurses as advocates, educators, and health promoters for patients and families.	Most EVALI cases were linked to vaping products containing THC and vitamin E acetate, though the exact etiology remained under investigation. The review highlights the rapid rise of e-cigarette use among youth, associated pulmonary risks, and the urgent need for nurses to act as educators, advocates, and health promoters in prevention and clinical management
4	2020	United States	Jun & Kim [42]	Cross-Sectional Study	To examine how universities communicate risks, policies, and cessation resources related to e-cigarettes on their websites, particularly under tobacco-free campus policies.	Among 581 U.S. universities, only 16% mentioned any e-cigarette risk, most often secondhand vaping (15.1%). While 80.9% of policies explicitly prohibited e-cigarettes, nearly one-fifth did not. Seventy percent noted policy impacts, mainly health protection (64%), and 60.6% described enforcement, more likely in Midwest (OR = 2.01, 95% CI: 1.16–3.47) and South (OR = 1.97, 95% CI: 1.16–3.33) vs. West, public institutions (OR = 1.80, 95% CI: 1.09–2.97), and campuses with housing (OR = 2.08, 95% CI: 1.18–3.64). Cessation resources were present in 55.1% of universities, but only 1.4% specific to e-cigarettes; these were more likely in 4-year (OR = 2.63, 95% CI: 1.18–5.85) and public institutions (OR = 2.50, 95% CI: 1.50–4.16). Off-campus resources appeared in 29.7%, again more likely in 4-year (OR = 2.97, 95% CI: 1.29–6.84) and public schools (OR = 2.34, 95% CI: 1.38–3.98). Overall, health risks were rarely communicated and fewer than half of universities listed cessation resources.
5	2020	Saudi Arabia	Natto [43]	Cross-Sectional Study	To evaluate dental students’ knowledge, education, and attitudes toward e-cigarettes, as well as their confidence in discussing them with patients.	Among 193 Saudi dental students (response rate 38.7%), 43.2% had ever used e-cigarettes and 5.7% were current users. Most ever users were also conventional smokers. Nearly all students (94.8%) reported insufficient education on e-cigarettes in the dental curriculum, and only 19.7% of never e-smokers felt confident discussing them with patients versus 40.5% of ever e-smokers (*p* = 0.035). Similarly, 30.9% of ever e-smokers believed e-cigarettes reduce cancer risk compared with 12.2% of never users (*p* = 0.013). Women were more likely to associate vaping with mood disorders (*p* = 0.025). Overall, knowledge of potential hazards was low (<30%), and 52.6% indicated they might recommend or were unsure about recommending e-cigarettes as cessation aids.
6	2020	Italy	Prigitano et al. [44]	Cross-Sectional Study	To investigate tobacco and e-cigarette use among health sciences university students and explore whether health-related education influences smoking cessation.	Among 560 Italian healthcare students, smoking prevalence was 34.8% (36.2% Italians vs. 21.4% foreigners, *p* = 0.02), with initiation at 16.4 years. E-cigarette experimentation was reported by 24.6% of students, including 44.6% of smokers, 38.6% of former smokers, and 9.4% of never smokers. Reasons for use were mainly smoking cessation (≈50%) or curiosity (48%), with preference for nicotine-free devices (64.5%). Over 44% of smokers were dual users, and 19.3% of former smokers quit via e-cigarettes. Smoking was more frequent among employed students (46.9% vs. 35.1%, *p* = 0.03) and those with smoking parents or friends (*p* < 0.01). A significant drop in smoking was observed only among Healthcare Assistance students between first and final year (33% vs. 0%, *p* = 0.033). Health education may both support quitting and prepare students to counsel patients.
7	2020	United States	Pulvers et al. [45]	Cross-Sectional Study	To describe the frequency of JUUL quit attempts among college users and identify factors associated with confidence in quitting and perceived difficulty.	Among 1001 undergraduates surveyed, 28.8% reported ever using JUUL, and nearly half of them (46.2%) were current users. Among current users, 40.5% had made at least one deliberate quit attempt. Overall, 47.8% of JUUL users reported attempting to quit, with prior attempts associated with a greater likelihood of perceiving quitting as difficult. Logistic regression analyses indicated that a shorter time to first JUUL use after waking was strongly associated with lower confidence in quitting (AOR = 0.02, 95% CI: 0.00–0.13, *p* < 0.001) and with greater perceived difficulty (AOR = 8.08, 95% CI: 2.15–30.35, *p* < 0.01). A history of quit attempts also predicted higher perceived difficulty (AOR = 5.97, 95% CI: 1.74–20.53, *p* < 0.01), although it was not related to confidence in quitting (*p* = 0.619). No demographic variables remained significantly associated with cessation perceptions after correction for multiple comparisons.
8	2020	New Zealand	Wamamili et al. [46]	Cross-Sectional (Prevalence) Study	To examine e-cigarette use, reasons for use, and harm perceptions among university students aged 18–24 in New Zealand.	In a survey of 1476 New Zealand university students, 40.5% reported ever using e-cigarettes, 6.1% were current users, and 1.7% vaped daily. Curiosity (67.4%) was the most common reason for use, followed by enjoyment (14.4%) and quitting smoking (2.4%). Overall, 76.1% of respondents perceived e-cigarettes as less harmful than tobacco cigarettes. Prevalence of ever use was significantly higher among males (51.2% vs. 33.1%, *p* < 0.001), Māori (57.1% vs. 39.2%, *p* < 0.001), and smokers (71.9% vs. 36.7%, *p* < 0.001) compared to their respective counterparts. Smokers were also more likely to report current use (13.7% vs. 5.1%, *p* < 0.001), daily use (5.6% vs. 1.2%, *p* < 0.001), and vaping to quit smoking (6.8% vs. 1.2%, *p* = 0.002). Younger students (18–20 years) were more likely than older peers (21–24 years) to cite curiosity as the main reason for vaping (74.7% vs. 56.1%, *p* < 0.001), whereas older students reported higher enjoyment (21.3% vs. 9.9%, *p* = 0.001) and longer daily use ≥1 month (16.5% vs. 8.1%, *p* = 0.008).
9	2020	China	Wang et al. [47]	Narrative Review	To estimate awareness and use of e-cigarettes among students at two universities in Shanghai and identify use-related factors and adverse effects.	In a cross-sectional survey of 869 students from two universities in Shanghai, awareness of e-cigarettes was very high (88.4%), with television advertisements (72.4%) and peers (41.2%) as the main sources of information. Ever use was reported by 4.6% and current use by 1.7%, with only 0.2% using daily. Multivariate analysis showed that cigarette smokers (AOR = 16.65, 95% CI: 7.10–39.05, *p* < 0.001) and students with peers who used e-cigarettes (AOR = 3.72, 95% CI: 1.59–8.70, *p* = 0.002) were significantly more likely to have tried e-cigarettes. Perceptions were predominantly favorable: 78% viewed them as healthier and 63.1% as less addictive than conventional cigarettes, while 69.5% believed they were less harmful and 81% considered them helpful for quitting smoking. Among ever users, the main reasons cited were reduced harm (55%) and smoking cessation (37.5%). Adverse effects were infrequently reported, most commonly thirst (10%) and throat irritation/cough (7.5%).
10	2021	-	Almeida-da-Silva et al. [48]	Cross-Sectional Study	To analyze the potential health effects of unregulated e-cigarette use on oral and systemic health.	E-cigarettes deliver toxic and carcinogenic compounds such as formaldehyde, acetaldehyde, and acrolein. Their heating mechanisms also release heavy metals (e.g., nickel, lead, cadmium) and nanoparticles into the aerosol, which can be inhaled and cause oral, pulmonary, and systemic health risks.
11	2021	Saudi Arabia	Alzahrani et al. [49]	Cross-Sectional Study	To assess knowledge and attitudes regarding the therapeutic use of e-cigarettes among undergraduate medical students.	Among 399 Saudi medical students (Mean age 21.8 years, 55.4% female), 36.6% had tried e-cigarettes and 11.5% were current users. Knowledge and attitudes were limited: 13.5% believed e-cigarettes were Food and Drug Administration-approved for cessation, 31.1% thought they reduced cancer risk, and 17.5% would recommend them to patients. While 35.9% agreed e-cigarettes were better than tobacco products, half (50.6%) recognized their addictive potential. Overall, only 23.6% expressed a favorable attitude toward their clinical use. Favorability was significantly higher among males (33.7% vs. 15.4% females, *p* < 0.001), smokers (34.2% vs. 19.4% nonsmokers, *p* = 0.004), ever e-smokers (36.3% vs. 16.2%, *p* < 0.001), and current e-smokers (52.2% vs. 19.8%, *p* < 0.001). In multivariate analysis, being male (OR = 2.39, 95% CI: 1.42–4.01) and ever e-smoking (OR = 2.25, 95% CI: 1.17–4.32) independently predicted favorable attitudes toward clinical use. Social media (77.4%) was the main source of information.
12	2021	United States	Ganson & Nagata [50]	Cross-Sectional Study	To examine the association between e-cigarette use and self-reported lifetime eating disorder diagnosis and risk among college students.	Among 51,231 U.S. college students from the Healthy Minds Study (2018–2019), 19.0% reported vaping in the past 30 days, 3.7% self-reported any lifetime eating disorder diagnosis, and 25.0% were at elevated risk for an eating disorder. Vaping was significantly more common among those with eating disorder pathology: 29.6% of vapers screened positive for elevated risk versus 23.9% of non-vapers, and 5.8% of vapers reported a lifetime eating disorder diagnosis versus 3.2% of non-vapers (all *p* < 0.001). Logistic regression analyses confirmed that vaping was associated with higher odds of self-reported eating disorder diagnoses: any eating disorder (AOR = 1.50, 95% CI: 1.27–1.77), anorexia nervosa (AOR = 1.39, 95% CI: 1.12–1.73), bulimia nervosa (AOR = 1.49, 95% CI: 1.14–1.94), binge-eating disorder (AOR = 1.72, 95% CI: 1.23–2.39), and elevated eating disorder risk (AOR = 1.11, 95% CI: 1.01–1.22), even after adjusting for demographics, mental health, alcohol, and cigarette smoking.
13	2021	United States	Jones et al. [51]	Cross-Sectional Study	To examine associations between self-efficacy, knowledge, depression, anxiety, and e-cigarette use among college students.	In a cross-sectional survey of 811 U.S. college students, 24.8% reported e-cigarette use, distributed as 7.0% daily users, 6.3% occasional users, and 11.5% infrequent users. Compared with non-users, e-cigarette users demonstrated significantly lower knowledge about associated risks, reduced self-efficacy, greater depressive symptoms, and poorer academic performance, although no differences were found for anxiety. Specifically, non-users reported higher knowledge scores (M = 28.66, SD = 3.30) than daily (M = 26.61, SD = 4.77) and occasional users (M = 27.33, SD = 4.22) [F(3,808) = 9.01, *p* < 0.001]. Self-efficacy was also greater among non-users (M = 31.85, SD = 5.04) compared with daily users (M = 29.01, SD = 8.87) [F(3,808) = 4.85, *p* < 0.01]. Occasional users showed higher depressive symptoms (M = 14.20, SD = 4.54) than non-users (M = 13.01, SD = 4.67) [F(3,808) = 8.31, *p* < 0.05]. Academic performance (GPA) was lower among daily users (M = 3.31, SD = 0.38) than non-users (M = 3.50, SD = 0.37) [F(3,808) = 5.49, *p* < 0.0001]. By contrast, no significant differences were observed for anxiety [F(3,808) = 1.00, *p* = 0.319].
14	2021	Qatar	Kurdi et al. [52]	Cross-Sectional Study	To assess the prevalence, knowledge, attitudes, and harm perceptions of e-cigarettes among university students in Qatar.	A survey of 199 students at Qatar University, 14% reported current e-cigarette use, with no significant gender differences (16.2% of males vs. 12.8% of females). Among users, 32% reported daily use, with pod-based devices (39.3%) and tank-based “mods” (32.1%) as the most common products. The Mean age of initiation was 20 years. Perceptions of reduced harm were prevalent: 67.9% of e-cigarette users (vs. 37.6% of non-users, *p* = 0.006) believed that e-cigarettes were less harmful than combustible cigarettes, and 78.6% of users (vs. 40.4% of non-users, *p* < 0.001) thought they could help prevent smoking traditional cigarettes. Nonetheless, knowledge gaps persisted: only 46.4% of users recognized e-cigarettes as a cause of lung cancer compared to 60.2% of non-users (*p* < 0.001), and average knowledge scores were significantly lower among users (M = 2.2, SD = 1.7) compared with non-users (M = 3.3, SD = 2.2; *p* = 0.041). Social influences played a key role. Having at least one close friend who smoked was strongly associated with e-cigarette use, with an adjusted odds ratio of 7.30 (95% CI: 2.39–22.25, *p* < 0.001). Users most frequently cited the absence of smell (85.7%), perceived reduced harm to themselves (75.0%) and to others (71.4%), ability to use in restricted places (60.7%), and availability of flavors (60.7%) as reasons for e-cigarette use. Stressful and social situations were the most common contexts for consumption
15	2021	United States	Newcombe [53]	Cross-Sectional Study	To identify reasons for JUUL use among college students.	A survey of 605 weekly JUUL users at a large U.S. university, four major categories of reasons for JUUL use emerged: self-help (48.4%), social (30.4%), experience (42.8%), and substance use/addiction (42.3%). Daily JUUL users were significantly more likely to report self-help motives (AOR = 1.66, 95% CI: 1.05–2.63) but less likely to endorse social reasons (AOR = 0.38, 95% CI: 0.23–0.63) compared with those who used JUUL 1–3 days per week. Gender and dependence patterns also shaped motivations: males were less likely than females to report self-help reasons (AOR = 0.63, 95% CI: 0.45–0.89) but more likely to cite experience-related motives such as flavor or buzz (AOR = 1.87, 95% CI: 1.32–2.65). Students with moderate or high dependence on JUUL were more likely to use for addiction-related reasons (AOR = 2.69, 95% CI: 1.56–4.62 and AOR = 2.41, 95% CI: 1.28–4.52, respectively). Notably, students who had never tried traditional cigarettes were over twice as likely to use JUUL for social reasons compared to cigarette-first users (AOR = 2.08, 95% CI: 1.22–3.54). Conversely, this group was less likely to cite substance use/addiction as a reason (AOR = 0.43, 95% CI: 0.25–0.74). Among ethnic groups, Asian students were less likely than non-Hispanic whites to report using JUUL for addiction-related reasons (AOR = 0.46, 95% CI: 0.23–0.92).
16	2021	United States	Omoike & Johnson [54]	Cross-Sectional Study	To investigate the prevalence and behavioral associations of e-cigarette use among college students, and identify predictive factors.	Among 498 college students (Mean age 20.9 years, 62.9% female, 76.5% non-Hispanic White), 43.2% reported ever using e-cigarettes. Multivariate logistic regression identified several behavioral and demographic predictors of vaping. Males had significantly higher odds of vaping compared to females (OR = 4.21, 95% CI: 1.61–11.01, *p* < 0.01). Students who used seat belts “most of the time” (vs. “always”) were more likely to vape (OR = 3.59, 95% CI: 1.09–11.76, *p* < 0.05). Conversely, students who reported texting/emailing while driving only 1–2 days per month had lower odds of vaping compared to those who texted daily (OR = 0.22, 95% CI: 0.05–1.00, *p* = 0.05). Other variables, such as age at first sexual intercourse, were not significantly associated with vaping status. Overall, the fully adjusted model was significant (*p* < 0.0001) and explained 67% of the variance in vaping behavior (Nagelkerke R^2^ = 0.67).
17	2021	United States	Oh et al. [55]	Cross-Sectional Study	To analyze psychotic experiences related to vaping using data from the Healthy Minds Study, conducted at 36 United-States universities.	Approximately 14–15% of students reported vaping in the past month. Logistic regression analyses demonstrated that vaping was significantly associated with psychotic experiences. In the unadjusted model controlling for sociodemographic variables, students who vaped were nearly twice as likely to report psychotic experiences (AOR = 1.88; 95% CI: 1.63–2.18; *p* < 0.001). This association remained significant after further adjustment for cigarette and marijuana use (AOR = 1.39; 95% CI: 1.17–1.64; *p* < 0.001), and persisted, although attenuated, when depression and anxiety were included in the model (AOR = 1.22; 95% CI: 1.03–1.45; *p* = 0.024).
18	2021	Chile	Páez et al. [56]	Cross-Sectional Study	To assess the prevalence, risk perception, motivations, and attitudes regarding e-cigarette use among medical students.	Among the 354 medical students surveyed, lifetime prevalence of e-cigarette use was substantially higher than recent use: 1.1% reported use in the past month, while 19.0% owned a device and 13.8% had used e-cigarettes to consume cannabis. The Mean age of initiation was 18.0 years (±2.2). In terms of perceptions, 37.1% believed e-cigarettes help people quit smoking, 39.7% perceived them as less harmful, and 19.0% viewed them as less addictive than conventional cigarettes. Risk perception was polarized: 26.6% reported moderate-to-severe risk in the short term, compared with 82.4% for long-term use. Positive perceptions (e.g., believing that e-cigarettes aid cessation or are less addictive) were significantly associated with both lifetime and past-year use (ORs ranging from 1.71 to 8.79, *p* < 0.05). Being male was strongly associated with e-cigarette use in the past year (OR = 8.15; 95% CI: 2.39–27.86). Current smokers showed markedly higher odds of e-cigarette use (lifetime OR = 7.13, 95% CI: 4.11–12.40; past-year OR = 8.79, 95% CI: 3.60–21.46). Motivations were mainly recreational, with the most frequent reasons being “just because” (91.3% among past-year users), “enjoying the flavor” (91.7%), and “relaxation” (47.8%). Less frequently endorsed motivations included harm reduction compared to cigarettes, perceived safety, lower cost, and appetite or weight control.
19	2021	Thailand	Phetphum et al. [57]	Cross-Sectional Study	To assess the prevalence and associated factors of e-cigarette use among university students in Northern Thailand.	Among 792 students surveyed, 18.1% reported past-30-day e-cigarette use. Most current users were male (73.4%), studied in non-health related faculties (94.4%), and initiated use after entering university (76.2%). The majority reported dual use of both e-cigarettes and conventional cigarettes (78.3%), with the most frequent contexts of use being during free time (40.6%) and while drinking alcohol (38.5%). Multivariable logistic regression identified six independent predictors of current e-cigarette use. Strongest associations were found for studying in non-health related faculties (AOR = 11.21; 95% CI: 4.88–25.71) and having friends who used e-cigarettes (AOR = 10.48; 95% CI: 5.96–18.41). Additional significant correlates included lower Grade Point Average (AOR = 1.93; 95% CI: 1.14–3.28), higher monthly income (AOR = 1.74; 95% CI: 1.09–2.78), perceiving e-cigarettes as less harmful than conventional cigarettes (AOR = 2.47; 95% CI: 1.50–4.07), and believing that using e-cigarettes in public is not illegal (AOR = 1.93; 95% CI: 1.19–3.15).
20	2021	France	Pougnet et al. [58]	Cross-Sectional Study	To study the prevalence of tobacco and e-cigarette use among health science students (Medicine, Dentistry, Physiotherapy, Nursing) at a university and university hospital in France.	The study reported a global vaping prevalence of 5.6% (74/1315), with substantial variation across disciplines, ranging from 2.4% among physiotherapy students to 10.4% among nursing students. Nursing students reported significantly higher e-cigarette use compared to their peers in other health disciplines (10.4% vs. 4.0%, *p* < 0.001). Moreover, vaping prevalence among nursing students increased markedly throughout their training, rising from 6.6% in first-year students to 18.9% in final-year students (*p* < 0.001). While dental students also showed a tendency toward higher e-cigarette use relative to other groups, this difference did not reach statistical significance (*p* = 0.09). Among the 74 students who reported vaping, 39% were exclusive users.
21	2021	United States	Rayman & Kessler [59]	Mixed Methods Study	To assess e-cigarette use, attitudes, and beliefs among university students, and inform nurse practitioners about the unique characteristics of young adult users.	Among 489 surveyed students, 18% reported vaping in the past 30 days, with higher prevalence among males (28.7%) and gender variant students (66.7%) compared with females (11.7%; χ^2^ = 27.82, *p* < 0.001). Vaping was also significantly associated with lower GPA (χ^2^ = 23.59, *p* < 0.001) and Greek life membership (30% vs. 18%; χ^2^ = 6.34, *p* = 0.012). Of current users, 41% vaped daily, and most initiated use between ages 15–20. The primary contexts of use were social events (72%) and with friends (60%). Students reported key motives including relaxation (71%), curiosity (20%), and smoking cessation (16%), though most also acknowledged negative health impacts (69%). The qualitative analysis (34 participants) identified five thematic categories: (1) safer than smoking—vaping perceived as a less risky alternative to cigarettes; (2) it’s cool in high school—strong social appeal during adolescence, diminishing in college; (3) generationally chill—vaping normalized within a tolerant peer culture; (4) ease of accessibility—products seen as cheap and easy to obtain despite age restrictions; and (5) quitting due to consequences—awareness of adverse effects such as headaches, mood swings, reduced athletic performance, and potential long-term health risks.
22	2021	-	Tarran et al. [60]	Narrative Review	To investigate the association between e-cigarette use and cardiovascular and respiratory diseases.	E-cigarettes contain oxidants, toxic metals, and carbonyl compounds associated with cardiovascular disease. Evidence also links use with asthma and other respiratory conditions. Although often posed as lower-risk alternatives to combustible smoking, current evidence cannot rule out negative cardiopulmonary effects, and the long-term safety of e-cigarettes remains uncertain.
23	2021	United States	Worthen & Ahmad [61]	Cross-Sectional Study	To analyze behaviors and patterns of multi-substance vaping among California college students.	Among California college students (*n* = 177), 68% reported single-substance vaping, while 32% engaged in multi-substance vaping. The most common substances combined with nicotine were flavorings (74%), marijuana (54%), and caffeine (23%). Multi-substance users reported significantly higher frequencies of vaping sessions per day compared to single-substance users (*p* < 0.01). Motivations for use were predominantly social reasons (62%) and the pursuit of desired psychoactive effects (45%). Students also highlighted experimentation and accessibility as contributing factors
24	2022	Jordan	AlMuhaissen et al. [62]	Cross-Sectional Study	To determine the prevalence of e-cigarette use among health sciences students at the University of Jordan and its correlation with sociodemographic factors, knowledge, and attitudes.	Among health sciences students at the University of Jordan, 37.4% reported ever using e-cigarettes and 19.7% were current users. Male students were significantly more likely to use e-cigarettes (AOR = 2.13, 95% CI: 1.45–3.14). Having a first-degree relative who used e-cigarettes was strongly associated with current use (AOR = 3.34, 95% CI: 2.13–5.23), as was having friends who vaped (AOR = 9.85, 95% CI: 6.07–15.96) and ease of access to e-cigarettes (AOR = 2.42, 95% CI: 1.60–3.68). Using social media as the primary source of information was also a significant predictor (AOR = 1.67, 95% CI: 1.13–2.48). Over 70% of participants correctly identified that e-cigarettes contain nicotine.
25	2022	Slovakia	Babjaková et al. [63]	Cross-Sectional Study	To assess e-cigarette use, perceived harm, and addiction risk among medical students in Slovakia.	Among medical students in Slovakia, 13.5% reported current e-cigarette use, with significantly higher prevalence among males (22.2% vs. 10.1% in females; OR = 2.53, 95% CI: 1.55–4.13) and foreign students (24.2% vs. 11.5% in Slovak students; OR = 2.44, 95% CI: 1.41–4.26). Prevalence was also strongly associated with smoking conventional cigarettes (46.9% among smokers vs. 8.1% among non-smokers; OR = 10.07, 95% CI: 5.85–17.34). Nearly 60% of respondents perceived e-cigarettes as less harmful than conventional cigarettes, particularly among those ≤24 years (61.8% vs. 51.5%; OR = 1.46, 95% CI: 1.03–2.07). Regarding perceived addictiveness, 41.2% considered e-cigarettes less addictive, 49% equally addictive, and 10% more addictive than tobacco cigarettes. Over half of students (55.6%) reported insufficient education on e-cigarettes and alternative tobacco products during medical training, with females more likely to express this view (58.8% vs. 47.5% in males; OR = 0.65, 95% CI: 0.49–0.85).
26	2022	United States	McLeish et al. [64]	Cross-Sectional Study	To examine college students’ knowledge about e-cigarettes, including ingredients, health risks, device modifications, and information sources.	The study surveyed 183 undergraduate students (Mean age = 19.98, SD = 1.98; 71.6% female) who had vaped in the past 30 days but had not smoked combustible cigarettes. Participants reported vaping on an average of 18.9 (SD = 11.3) days in the last month, with 43.2% identifying as daily users. The mean Penn State Electronic Cigarette Dependence Index score was 9.38 (SD = 5.10), reflecting a moderate dependence level. Regarding knowledge, most students recognized cardiovascular (77.0%) and pulmonary (89.1%) risks, but less than half identified seizures (43.7%) or depression (47%) as health effects. Ingredient knowledge was limited: only 34.4% identified formaldehyde, 29.5% benzoic acid, 29.0% volatile organic compounds, 43.2% heavy metals, and 45.9% propylene glycol. Concerning device use, 6.6% had modified the voltage of their e-cigarette at least once, and 21% individualized their devices most to all of the time. Google (39.3%) was the primary information source, followed by friends (27.9%), doctors or other medical professionals (17.5%), and vape shop employees (14.8%). No significant differences were found in knowledge or behaviors by gender, race, or year in school (all *p* > 0.05).
27	2022	Germany	Seidel et al. [65]	Cross-Sectional Study	To analyze whether e-cigarette use predicts future cannabis experimentation among German youth.	This two-wave prospective study followed 3040 cannabis-naïve students (Mean age = 14.8, SD = 0.93; 44.5% male) from 74 secondary schools in North Rhine-Westphalia, Germany, over 18 months. At baseline, 33.8% reported lifetime e-cigarette use. During follow-up, 17.4% initiated cannabis use. Cannabis initiation was significantly higher among baseline e-cigarette users compared with non-users (34.5% vs. 10.4%; χ^2^ = 250.34, *p* < 0.001). Multilevel Poisson regression adjusted for demographic and behavioral covariates showed that baseline e-cigarette use predicted later cannabis initiation (ARR = 1.83, 95% CI = 1.48–2.25). Other significant predictors included male sex (ARR = 1.50, CI = 1.26–1.79), higher sensation-seeking (ARR = 1.28, CI = 1.09–1.49), peer cannabis use (ARR = 1.89, CI = 1.62–2.20), cigarette use (ARR = 1.71, CI = 1.39–2.10), and alcohol use (ARR = 3.12, CI = 2.17–4.50). Interaction analyses revealed that the strength of the association between e-cigarette use and cannabis initiation was moderated by conventional cigarette use (ARR = 0.48, CI = 0.37–0.64) and sensation seeking (ARR = 0.77, CI = 0.61–0.97). The difference in cannabis initiation rates between e-cigarette users and non-users was larger among never smokers (13.7 percentage points) than ever smokers (4.4 percentage points), and among lower sensation seekers (11.8 points) compared with higher sensation seekers (9.8 points).
28	2023	United States	Hair et al. [66]	Cross-Sectional Study	To evaluate an online prevention curriculum aimed at increasing knowledge about the dangers of vaping among at-risk youth.	A total of 1399 middle and high school students completed pre- and post-intervention assessments. Participation in the online curriculum produced a significant improvement in knowledge scores (mean increase = 3.24, *p* < 0.001). Regression analyses indicated that the effect was most pronounced among students with the lowest baseline knowledge (β = 5.84, SE = 0.03), suggesting the program was especially effective in reducing knowledge gaps.).
29	2023	United States	Holden & Simerson [67]	Quasi-experimental study	To train nurses in the SBIRT technique as a tool to combat the vaping epidemic among university students.	This quasi-experimental study evaluated the impact of an educational intervention to train nurses in the SBIRT (Screening, Brief Intervention, and Referral to Treatment) technique as a strategy to address the vaping epidemic among college students. The sample included 28 nursing students from a U.S. university. At baseline, 71.4% reported no prior experience with motivational interviewing. Following the intervention, participants demonstrated significant improvements in both knowledge and self-efficacy scores (knowledge: pre-test M = 6.71, SD = 1.48 vs. post-test M = 9.29, SD = 0.98, *t* = −9.88, *p* < 0.001; self-efficacy: pre-test M = 61.89, SD = 10.54 vs. post-test M = 76.82, SD = 9.21, *t* = −7.98, *p* < 0.001). Moreover, 85% of participants stated they would recommend SBIRT training to other nursing professionals. These results underscore the potential of SBIRT-based curricula to enhance nurses’ competencies in addressing vaping behaviors among young adults.
30	2023	Thailand	Kaewsutha & Karawekpanyawong [68]	Cross-Sectional Study	To examine the prevalence of tobacco and e-cigarette use among Thai dental students, along with their attitudes toward tobacco control and educational exposure.	This cross-sectional survey included 1968 Thai dental students (response rate = 42.8%). The prevalence of current tobacco or e-cigarette use was 4.2%, with e-cigarettes accounting for 95% of use (4.0%) compared with 1.4% for conventional cigarettes. Male students were significantly more likely than females to report ever use (33.9% vs. 12.8%, *p* < 0.001) and current use (10.4% vs. 1.7%, *p* < 0.001). Among current users (*n* = 82), 58.5% reported exclusive e-cigarette use, and 36.6% used more than one tobacco product. Most students expressed positive attitudes toward tobacco control: 85.1% agreed health practitioners should act as role models, and 95.4% endorsed routinely advising patients to quit smoking. However, only 39.3% had received training on cessation counseling, and 40.4% on tobacco cessation aids. Attitudes toward e-cigarettes were predominantly negative, with 60.5% strongly disagreeing that e-cigarettes are non-addictive and 45.8% disagreeing they reduce health risks compared to cigarettes
31	2023	Philippines	Resano et al. [69]	Cross-Sectional Study	To evaluate the association between smoking, e-cigarette use, and perceived stress levels among nursing students in Manila, Philippines.	This cross-sectional study surveyed 249 nursing students in Manila (Mean age = 19.4, SD = 0.9; 77.9% female). Most were never users (61.0%), while 4.0% were exclusive cigarette smokers, 12.9% exclusive e-cigarette users, and 22.1% dual users. Overall, 16.1% reported high perceived stress levels (Perceived Stress Scale >26). In adjusted analyses, dual users were more than twice as likely to report high stress compared with never users (APR = 2.47, 95% CI = 1.29–4.73, *p* < 0.01). Exclusive cigarette smokers showed a similar trend (APR = 2.16, 95% CI = 0.89–5.26, *p* < 0.10), while exclusive e-cigarette users did not differ significantly (APR = 0.71, 95% CI = 0.22–2.25). Peer and family use were common (>80%) but not significantly associated with stress.
32	2023	United Kingdom	Vilcassim et al. [70]	Cross-Sectional Study	To characterize patterns of e-cigarette device use among university students, focusing on sex-based differences.	This cross-sectional survey included 394 students aged 18–24 years at the University of Alabama at Birmingham, of whom 61 were exclusive e-cigarette users, yielding a prevalence of 15.5%. The most common device type was disposable e-cigarettes (47%), followed by tanks/mods (19%), rechargeable e-cigarettes/Blu (17%), and Juul (12%). Device preferences differed significantly by sex (χ^2^, *p* < 0.05): female users predominantly reported disposables/Juul, whereas males more frequently used tanks/mods and rechargeable devices. Weekly frequency of use also varied, with females more likely to report 1–3 days/week and males 6–7 days/week. In logistic regression adjusted for place of residence, males had five times higher odds of using tanks/mods or rechargeable devices compared with females (AOR = 5.01, 95% CI = 1.37–18.0). No significant associations were found for race, field of study, or year in college.
33	2024	-	Albadrani et al. [71]	Systematic Review	To estimate the global prevalence of ENDS use among school and university students.	This systematic review and meta-analysis synthesized data from 146 studies comprising 4,189,145 students across 53 countries. The pooled current prevalence of ENDS use was 10.2% (95% CI: 9.5–11.0%, *p* < 0.001), with higher rates in males (10.2%, 95% CI: 8.9–11.5%) compared to females (7.5%, 95% CI: 6.5–8.4%). The lifetime prevalence was estimated at 22.0% (95% CI: 20.0–24.0%), also higher among males (27.7%) than females (23.7%). Subgroup analyses by continent showed current prevalence ranging from 4.0% in South America to 12.7% in Europe, while lifetime prevalence was highest in North America (26.8%). Significant heterogeneity was observed across studies (I^2^ > 99%, *p* < 0.001), and Egger’s test indicated publication bias (*p* < 0.001).
34	2024	United States	Bataineh et al. [72]	Cross-Sectional Study	To examine problematic social media use and exposure to e-cigarette-related content among Mexican-American college students.	This ecological momentary assessment (EMA) study included 51 Mexican-American college students (Mean age = 21.0, SD = 1.73; 72.5% female) across five public universities in Texas. Over a 14-day monitoring period, participants reported an average of 4.6 h/day of social media use; those with problematic social media use spent significantly more time (5.8 vs. 4.3 h/day; *p* < 0.05). Nearly 50% reported exposure to e-cigarette-related content on social media, most frequently on TikTok, with 18.4% originating from influencers. E-cigarette use was common, with students reporting use on 5.7 of 14 days on average; 39.2% used on ≥7 days, while only 2.4% used daily. Disposable devices (e.g., Elf Bar, Escobar) and fruit or menthol flavors predominated. Regression analyses adjusting for age, sex, and SES revealed that problematic social media use was positively associated with the number of days e-cigarettes were used (β = 0.14, 95% CI: 0.07–0.20, *p* < 0.001), frequency of daily use (β = 0.03, CI: 0.02–0.05, *p* < 0.001), and nicotine concentration consumed (β = 0.08, CI: 0.03–0.14, *p* = 0.004).
35	2024	United States	Folivi et al. [73]	Cross-Sectional Study	To examine the associations between need frustration, ruminative thinking, frequency of e-cigarette use, and nicotine dependence among college users.	This cross-sectional study analyzed data from 610 college e-cigarette users to examine the associations between basic psychological need frustration, ruminative thinking, frequency of e-cigarette use, and nicotine dependence. Bivariate correlations showed that all three types of need frustration—autonomy (r = 0.47, *p* < 0.001), competence (r = 0.46, *p* < 0.001), and relatedness (r = 0.49, *p* < 0.001)—were positively associated with ruminative thinking, frequency of use, and e-cigarette dependence. Structural equation modeling confirmed that need frustration predicted greater rumination, which in turn mediated its relationship with nicotine dependence (indirect effects significant at *p* < 0.001). The final model demonstrated good fit (χ^2^(146) = 302.19, *p* < 0.001; CFI = 0.95; TLI = 0.94; RMSEA = 0.045, 90% CI = 0.038–0.052), supporting the proposed pathways.
36	2024	France	Kinouani et al. [74]	Cross-Sectional Study	To describe the transition from cigarette smoking to exclusive or partial e-cigarette use among French university students using a mixed-methods approach.	This mixed-methods study (Electra-Share project) analyzed e-cigarette use among students at the University of Bordeaux. In the weighted online sample (*n* = 415), 41% had ever tried e-cigarettes, but only 7.1% (95% CI: 4.2–12.0) were current users (past 30-day). Current vaping was significantly associated with smoking status (χ^2^, *p* < 0.001): 79.5% of current vapers were also current smokers, 18.2% were former smokers, and only 2.3% were never-smokers. Among campus participants (*n* = 211), 91% used nicotine e-liquids, and frequency of use differed by smoking status (χ^2^, *p* < 0.001): daily vaping predominated among current (76.8%) and former smokers (92.5%) compared to non-smokers (46.7%). Main reasons for trying e-cigarettes included quitting smoking (72.5% of former smokers; 61.9% of current smokers), lower cost, and reduced perceived harm. Qualitative interviews (*n* = 30) revealed that most began vaping out of curiosity, but continued use required personal commitment (e.g., device purchase, acquiring technical skills). Integrative analysis identified two broad profiles: (1) dual users, who supplemented smoking with vaping, and (2) exclusive vapers (former smokers), some of whom adopted vaping as a long-term identity.
37	2024	Pakistan	Maqsood et al. [75]	Cross-Sectional Study	To analyze tobacco and e-cigarette use among university students and assess their awareness of health initiatives and cessation efforts.	In this large cross-sectional survey of 683 university students across Pakistan, 16.8% reported ever using e-cigarettes, while 23.6% had smoked cigarettes and 20.4% had tried shisha (*p* < 0.001 for all, Binomial test). Among e-cigarette users, only 3.1% reported daily use, with the majority using occasionally (10.4%). Social acceptance was a key driver, as 58.3% of respondents perceived smoking or vaping as socially acceptable (*p* < 0.001). Awareness levels showed mixed results: while 59.3% were aware of university policies on tobacco/vaping (*p* < 0.001), only 46.6% knew of smoking cessation or anti-vaping programs (*p* < 0.001). Concern about health risks was high, with 74.5% acknowledging long-term harms of vaping (*p* < 0.001). Logistic regression showed that male students were more likely to be policy-aware (OR = 1.67, 95% CI: 1.11–2.49, *p* = 0.013), and awareness was significantly higher among dentistry (OR = 3.37, 95% CI: 1.82–6.23, *p* < 0.001) and medical students (OR = 1.89, *p* = 0.016) compared to other fields.
38	2024	Egypt	Mostafa & Taha [76]	Cross-Sectional Study	To report the prevalence of e-cigarette use among medical students at Cairo University.	A cross-sectional study conducted among 300 medical students at Cairo University, the prevalence of e-cigarette use was reported at 7.3%, lower than that of conventional cigarettes (14%) and shisha (12.7%). Use was significantly more common among clinical-year students compared to preclinical students (11.3% vs. 3.3%, *p* = 0.008). When considering combined e-cigarette and POD use, the prevalence reached 8.3%, again higher among clinical students (12% vs. 4.7%, *p* = 0.02). Multivariable analyses identified being in the clinical phase of study, cigarette or shisha smoking, and having friends who vape as independent predictors of e-cigarette use. Knowledge about e-cigarettes was widespread (88.3%), with media (41.8%) and friends (37.5%) cited as the main sources of information. Compared to non-users, e-cigarette users were significantly less likely to believe that these products are addictive or cause respiratory problems, and more likely to perceive them as less harmful, less nicotine-containing, and potentially helpful for smoking cessation.
39	2024	United States	Roh [77]	Cross-Sectional Study	To identify predictors of e-cigarette use among Hispanic university students in Texas (United States).	Among 316 undergraduate students, 33.9% were current e-cigarette users, 29.1% former users, and 37.0% never users. Hispanic participants were more likely to report prior vaping experience compared to White students (AOR = 2.42, 95% CI: 1.00–5.84). Current vaping was predicted by being upper-level (junior/senior) students (AOR = 2.47, 95% CI: 1.24–4.91) and by prior use of other tobacco products (AOR = 6.80, 95% CI: 3.90–11.8). The most frequent reasons for current use were to achieve a nicotine “buzz” (49.5%) and to cope with stress or negative emotions (47.7%). Quit attempts were reported by 74.3% of current users, with women significantly more likely than men to attempt quitting in the past year (80.3% vs. 54.2%, AOR = 3.72, 95% CI: 1.20–11.6). Hispanic users also reported a higher average number of quit attempts than White students (4.36 vs. 3.15, adjusted β = 1.62, 95% CI: 0.23–3.00).
40	2024	United States	Singer et al. [78]	Longitudinal Cohort Study	To examine the relationship between patterns of nicotine salt-based e-cigarette use and symptoms of nicotine dependence in a university cohort.	Among 411 JUUL ever-users, 75.8% reported use in the past 30 days, with 37.4% vaping on 6–30 days and 29.6% finishing a pod within ≤1 week. Higher frequency of use (≥6 vs. 0–5 days) significantly predicted nicotine dependence symptoms, as measured by the E-cigarette Dependence Scale (AOR = 4.93; 95% CI: 1.64–14.83; *p* = 0.005) and the Wisconsin Smoking Withdrawal Scale Craving subscale (AOR = 5.82; 95% CI: 1.66–20.35; *p* = 0.006). Quantity of use (≤1 week vs. >1 week to finish a pod) was also associated with dependence, predicting symptoms on the E-cigarette Dependence Scale (AOR = 3.00; 95% CI: 1.15–7.84; *p* = 0.025), the Hooked on Nicotine Checklist (AOR = 3.66; 95% CI: 1.35–9.94; *p* = 0.011), the Wisconsin Smoking Withdrawal Scale Anger subscale (AOR = 3.34; 95% CI: 1.22–9.11; *p* = 0.012), and the Wisconsin Smoking Withdrawal Scale Craving subscale (AOR = 6.15; 95% CI: 1.96–19.25; *p* = 0.002). Both frequency and quantity of e-cigarette use were positively associated with subsequent nicotine dependence.
41	2024	United States	Ou et al. [79]	Prospective Cohort Study	To examine whether reasons for e-cigarette use predict higher or lower levels of dependence among college students.	A prospective cohort study with 366 undergraduate students from three U.S. universities (Mean age = 19.9 years; 48% male; 68% White). Eligible participants had used e-cigarettes at least weekly in the past month, and follow-ups spanned four semesters (2019–2023). Dependence was measured using the Penn State Electronic Cigarette Dependence Index (PSECDI). Results showed that vaping for relaxation (β = 0.63, *p* < 0.05) and taste (β = 0.63, *p* < 0.05) were significantly associated with higher dependence, while experimentation predicted lower dependence (β = −1.21, *p* < 0.001). Dependence was also higher among students reporting greater nicotine concentrations (β = 0.38, *p* < 0.001), early-onset cigarette use (β = 2.62, *p* < 0.01), established smoking history (β = 1.86, *p* < 0.01), and concurrent tobacco or alcohol use (*p* < 0.05). Male (β = −1.76, *p* < 0.001) and Hispanic (β = −1.66, *p* < 0.01) students exhibited significantly lower dependence.
42	2025	Croatia	Kajan et al. [80]	Cross-Sectional Study	To assess knowledge, attitudes, and use of e-cigarettes among Croatian nursing students, including their perspectives on nurses’ roles in cessation counseling.	A cross-sectional study among 1039 Croatian nursing students (Mean age = 27 years; 89% female) from 10 higher education institutions. An online questionnaire, adapted and validated from prior studies, assessed sociodemographic data, smoking and e-cigarette use, knowledge (0–5 score), and attitudes toward cessation and professional roles. Overall, 43% were current smokers, 12% former smokers, and 45% never smokers. More than half reported current e-cigarette use—76% recreationally and 24% for cessation. Notably, 60% had never received formal education on smoking cessation, and only 0.2% answered all knowledge questions correctly. Confidence in advising smokers was very low (12%), and two-thirds (66%) could not advise on e-cigarettes. Most participants (72%) agreed that nurses should help patients quit, 68% emphasized the need for further training, and 52% supported nurses as role models by remaining smoke-free. Logistic regression showed that smoking status (OR = 3.73, *p* < 0.001) and younger age (OR = 0.97, *p* = 0.009) predicted counseling confidence, while gender and formal education were not significant predictors.
43	2025	-	Soerianto & Jaspers [81]	Narrative Review	To analyze the pathogenesis, diagnosis, and treatment challenges of EVALI.	This narrative review synthesized evidence on the etiology, diagnosis, and treatment of EVALI, drawing from PubMed, Centers for Disease Control and Prevention, and Food and Drug Administration reports. Vitamin E acetate, detected in 94% of bronchoalveolar lavage samples in a multi-state Centers for Disease Control and Prevention study and present in 81% of THC-containing products tested by the Food and Drug Administration, emerged as the main toxicant, although other agents may also contribute. Diagnosis remains one of exclusion, often confounded with pneumonia or COVID-19. Corticosteroids led to clinical improvement in approximately 80% of cases. Despite a decline after regulatory bans, new cases continue to be reported, underscoring persistent risks and the need for stricter control.

**Abbreviations:** AOR: Adjusted Odds Ratio; APR: Adjusted Prevalence Ratio; ARR: Adjusted Relative Risk; CAP: Community-Acquired Pneumonia; CDC: Centers for Disease Control and Prevention; CFI: Comparative Fit Index; CI: Confidence Interval; COVID-19: Coronavirus Disease 2019; EDS: E-cigarette Dependence Scale; ENDS: Electronic Nicotine Delivery Systems; EVALI: E-cigarette or Vaping-Associated Lung Injury; FDA: Food and Drug Administration; F (e.g., F(3,808)): Fisher’s F statistic (ANOVA); GPA: Grade Point Average; HONC: Hooked on Nicotine Checklist; I^2^: Higgins’ I-squared (measure of heterogeneity in meta-analysis); JUUL: Registered trademark of JUUL Labs, Inc.; M: Mean; OR: Odds Ratio; POD: Pod-based e-cigarette device; PSECDI: Penn State Electronic Cigarette; Dependence Index; R^2^: Coefficient of Determination; RMSEA: Root Mean Square Error of Approximation; SD: Standard Deviation; SE: Standard Error; SES: Socioeconomic Status; SBIRT: Screening, Brief Intervention, and Referral to Treatment; THC: Tetrahydrocannabinol; TLI: Tucker–Lewis Index; U.S.: United States.

#### 3.3.1. Patterns and Prevalence of E-Cigarette Use

E-cigarette use among university students has become a growing public health concern, with patterns of initiation and prevalence revealing notable regional and demographic variations. Multiple studies report that initiation most frequently occurs during the transition to university, a period marked by increased autonomy, reduced parental supervision, and intensified exposure to peer influence. For example, among Thai students, 76.2% reported initiating e-cigarette use in their first year at university, while only 23.8% started during secondary school [52,56,57]. In Chile, the mean age of initiation was 18 years (±2.2), and in a Qatar university sample it was slightly higher, at 20.4 years (±8.2) [52,56].

Globally, the estimated prevalence of e-cigarette use among students ranges widely. A recent systematic review involving over 4 million participants from 53 countries reported a pooled prevalence of 10.2%, with Europe showing the highest prevalence (12.7%) and South America the lowest (4%) [71]. Individual studies highlight considerable heterogeneity: in the United States, some samples report rates as high as 43.2% [54]; in Chile, although 32.9% had tried e-cigarettes at least once, only 6.8% reported use in the past year and 1.1% in the past month [56]. Similarly, high rates are found in the Middle East, with prevalence estimates of 37.1% in Jordan [62], 14% in Qatar [52], and 7.3% in Egypt [76]. In Asia, prevalence in Thailand reached 18.1% among university students, although it dropped to 4.2% in specific disciplines such as Dentistry [57,68].

In Europe, figures range from 13.5% in medical students from Slovakia [63], to 5.6% in a French health sciences sample [58], and drop significantly in Spain, where only 0.4% of students reported exclusive use of e-cigarettes and 0.3% reported dual use with traditional tobacco [40]. These variations likely reflect differing national regulations, access and affordability, public perceptions of harm, and socio-cultural norms.

Disciplinary background also appears to influence prevalence. While some studies report higher use among medical students—with estimates ranging from 13.3% to 37%, depending on country and year of study [56,62,63,76]—other research highlights elevated rates among nursing students (10.4% in France, 30.7% in Jordan) [58,62] and pharmacy students (24% in Jordan) [62]. Prevalence varies even within the same discipline: dental students reported use rates of 4.2% in Thailand and 42.9% in Jordan [62,68], suggesting potential methodological, cultural, or educational disparities.

Sex differences are also commonly reported. Most studies identify higher prevalence among male students, with the global estimate showing 10.2% use in men versus 7.5% in women [71]. In Chile, lifetime prevalence was 37.6% in men and 28.3% in women, with a more pronounced gap in recent use [56]. However, in the Appalachian sample, 62.9% of participants were female, whereas lifetime vaping prevalence reached 43.2% [54]—suggesting that sex-specific interpretations require caution. This may reflect culturally mediated patterns or region-specific sociobehavioral norms. Device preference has also been explored, with several studies indicating a predominance of disposable e-cigarettes—particularly among younger users—with prevalence estimates ranging from 45% to 73% [70,72]. This trend carries important regulatory implications, as disposable devices often deliver higher nicotine concentrations and are typically more affordable and accessible.

Taken together, the evidence highlights a highly heterogeneous pattern of e-cigarette use among university students, shaped by geographical region, disciplinary background, and gender. While prevalence is consistently higher in North America and parts of the Middle East, it remains comparatively lower in Southern Europe. These differences appear to reflect a combination of contextual factors, including regulatory environments, cultural perceptions of harm, and affordability of devices. Disciplinary variations suggest that health sciences training may partially mitigate use, although findings among nursing and dental students reveal important exceptions. Similarly, although males generally report higher prevalence, female use is not negligible and, in some regions, surpasses male use, pointing to the need for gender-sensitive analyses. Overall, the synthesis indicates that prevalence is not uniform but strongly conditioned by socio-cultural and educational contexts, underscoring the necessity of locally tailored prevention strategies.

#### 3.3.2. Behavioral and Social Determinants of E-Cigarette Use

The initiation and persistence of e-cigarette use among university students are shaped by a complex interplay of behavioral, social, and contextual factors. A consistent finding across studies is the prominence of curiosity and peer influence as primary drivers of initial use. In surveys conducted in New Zealand and Shanghai, 67.4% and 55% of participants, respectively, cited curiosity as the main motivation [46,47]. Similarly, a substantial proportion of students reported having close friends who used e-cigarettes (up to 80%), and nearly 10% had a first-degree relative who used them, underscoring the role of social normalization in shaping usage behavior [43,44].

Other frequently reported motives include relaxation, emotional self-regulation, and facilitated socialization. In United States-based studies, particularly among Hispanic students, vaping was often linked to stress relief, anxiety, and depressive symptoms [53,59,77]. The desire to experience a nicotine buzz, attractive flavors, and the ability to use e-cigarettes discreetly in restricted areas were also cited as contributing factors [52,56,61].

Academic and structural variables also play a role. In Thailand, higher e-cigarette use was observed among students in non-health-related faculties, those with lower academic performance, and individuals with above-average monthly income [57]. Additionally, e-cigarette use was reported to be embedded in daily routines, with common usage contexts including leisure time, alcohol consumption, upon waking, and during meals.

Sociodemographic variables such as sex and parental education show mixed associations. While most studies indicate a higher prevalence among men, some research from the United States identified greater female participation, although men remained more likely to use e-cigarettes overall [54,70]. In certain Middle Eastern contexts, a correlation was found between e-cigarette use and having parents with higher educational attainment, particularly in health-related disciplines [62].

Digital environments have also emerged as powerful influences. Exposure to pro-vaping content on social media platforms—particularly TikTok and Instagram—has been linked to increased e-cigarette use among students, with roughly 50% of respondents reporting having viewed such content, often produced by influencers or industry-sponsored accounts [72].

Some studies have explored less conventional associations. For instance, certain behaviors such as inconsistent seatbelt use or texting while driving have been proposed as proxy indicators of broader risk-taking profiles associated with e-cigarette use, suggesting that vaping may co-occur with impulsivity and sensation-seeking traits [54].

The potential role of e-cigarettes as a smoking cessation aid was frequently cited among users. In universities in Texas and Bordeaux, over 70% of current users were recent former smokers, and more than half cited the lower cost of e-cigarettes as an added motivation [74,77]. However, concerns remain regarding the actual efficacy of e-cigarettes in promoting sustained cessation, particularly in cases of dual use or persistent dependency.

Recent studies have also examined psychological correlates of vaping. For example, students with high levels of autonomy frustration, ruminative thinking, and maladaptive coping strategies were more likely to report frequent e-cigarette use and signs of psychological dependence, even in the absence of peer pressure or external triggers [73]. These findings suggest that e-cigarette use may function as both a coping mechanism and a socially mediated behavior.

In summary, the behavioral and social determinants of e-cigarette use among university students converge around three major drivers: social normalization, psychological regulation, and structural opportunities for access. Curiosity and peer influence consistently emerge as primary triggers for initiation, whereas stress, emotional self-regulation, and the pursuit of social belonging sustain continued use. Although patterns vary across cultural contexts, with academic discipline and socioeconomic background shaping prevalence in distinct ways, the overall synthesis suggests that vaping is deeply embedded within students’ social and emotional lives. Digital exposure further amplifies these dynamics, highlighting the interplay between offline peer networks and online content. Collectively, these findings underscore the need for interventions that not only address individual behaviors but also target broader social environments and digital ecosystems where e-cigarette use is normalized.

#### 3.3.3. Knowledge and Beliefs About E-Cigarettes

University students’ knowledge and beliefs about e-cigarettes critically shape their perceptions of risk, intentions to use, and receptiveness to health education. The reviewed literature reveals widespread information gaps, misperceptions, and a predominance of informal sources, even among students in health-related disciplines.

Regarding health risks, most students are aware that e-cigarette use may cause pulmonary and cardiovascular damage, but awareness of less visible risks—such as seizures, depression, or toxic exposure—is markedly lower. In a United Stated-study, only 43.7% of students identified the risk of seizures, and 47% recognized the association with depression [64]. In Qatar, just 58.3% acknowledged the risk of lung cancer, and over 50% were unaware that e-cigarettes may contain carcinogenic substances [52].

Notably, health sciences students are not exempt from these deficits. In Jordan, 72.3% of health students correctly identified e-cigarettes as carcinogenic, and 51.5% acknowledged their addictive potential, but only 10.8% knew that e-cigarettes are not approved by the Food and Drug Administration [62]. Similarly, in Croatia, nursing students who were e-cigarette users demonstrated slightly greater awareness of health risks—particularly regarding the lack of evidence for e-cigarettes as cessation tools—than their non-using peers [80].

Erroneous beliefs persist, especially regarding e-cigarettes’ comparative safety and therapeutic value. In Chile, 39.7% of students believed e-cigarettes to be less harmful than traditional cigarettes, and 19% believed them to be less addictive [56]. In Saudi Arabia, 17.5% of medical students said they would recommend e-cigarettes to help patients quit smoking [49]. In Slovakia, nearly 60% of medical students held similar beliefs, and more than half considered their university education on the topic to be insufficient [63]. Likewise, in a Saudi Arabia dental program, 95% of students reported feeling unprepared to advise patients about e-cigarettes [43].

When examining knowledge of chemical components, fewer than 50% of students correctly identified key toxicants such as formaldehyde (34.4%), benzoic acid (29.5%), or heavy metals (43.2%) [64]. Such findings reveal not only a lack of familiarity with e-cigarette ingredients but also a limited understanding of their physiological implications.

Students primarily rely on non-professional sources for information. Across several studies, the most frequently cited sources were peers, social media platforms, and search engines like Google, while only 17.5% reported consulting healthcare professionals [39,64,72]. Exposure to e-cigarette -related content on Instagram, TikTok, or YouTube was high, with 50% of participants having encountered such material, often produced by influencers or user-generated content (65.8% and 18.4%, respectively) [72].

Institutional awareness was also limited. In one United States-study, 59.3% of students were aware of their university’s tobacco and e-cigarette policy, but only 46.6% were aware of any cessation programs available on campus or through university social media [75].

Overall, the literature reveals a striking mismatch between students’ widespread exposure to e-cigarettes and their limited, often inaccurate, knowledge of associated health risks. Misperceptions regarding safety, addictive potential, and therapeutic value persist even among health sciences students, reflecting gaps in formal education and insufficient institutional engagement. The reliance on peers, social media, and user-generated content as primary information sources further perpetuates misinformation, overshadowing professional and educational channels. While some subgroups, such as nursing or medical students, demonstrate slightly greater awareness, these exceptions do not mitigate the broader trend of fragmented and unreliable knowledge. Taken together, these findings highlight the urgent need for evidence-based health communication strategies that prioritize clarity, credibility, and accessibility across both academic curricula and digital platforms.

#### 3.3.4. Health Consequences of E-Cigarette Use

Although originally promoted as a safer alternative to conventional tobacco, e-cigarettes have been associated with a wide range of adverse health outcomes, spanning pulmonary, cardiovascular, neurological, and psychosocial domains. The health risks stem primarily from the inhalation of nicotine, aldehydes, heavy metals, and various chemical solvents, many of which are unregulated and potentially toxic, especially when delivered through high-concentration nicotine salts and flavored aerosols.

##### Addiction and Nicotine Dependence

The majority of e-cigarette liquids contain nicotine, a highly addictive substance whose addictive potential has been equated to that of heroin or cocaine [41]. Acute nicotine intoxication (“nic-sick”) has been reported, with symptoms such as nausea, headaches, palpitations, and dizziness. The use of high-nicotine delivery devices (e.g., JUUL or disposable pods) has been particularly linked to moderate-to-severe dependence, especially when consumption is motivated by stress relief or enjoyment of flavors [78,79].

##### Association with Risk Behaviors

E-cigarette use has been consistently associated with polysubstance use and risk-taking behaviors. E-cigarette use has also been identified as a gateway behavior to conventional cigarette smoking and cannabis use, particularly among young adults with no prior history of substance use [65].

##### Respiratory and Cardiovascular Impacts

One of the most serious e-cigarette-related conditions is EVALI, a syndrome characterized by severe respiratory symptoms, acute lung injury, and sometimes fatal outcomes. As of 2020, over 2800 cases had been reported in the United States, with a third occurring in individuals under 25 years of age [41,81]. Although its pathogenesis remains multifactorial, EVALI has been linked to THC-adulterated cartridges and vitamin E acetate. The clinical picture often includes respiratory distress, gastrointestinal symptoms, and systemic inflammation, and treatment typically involves corticosteroids and supportive care.

Cardiovascular consequences have also been documented. Exposure to propylene glycol, nicotine, and heavy metals in e-cigarette aerosols may impair vascular function, evidenced by alterations in arterial stiffness markers such as pulse wave velocity and aortic pressure [60]. These findings suggest a risk of early arterial damage, even in young, otherwise healthy users.

##### Oral Health and Microbiome Alterations

Studies have shown that e-cigarette use alters the oral microbiome, favoring bacterial dysbiosis and increasing the risk of periodontal disease and dental caries. Users exhibit a higher abundance of pathogenic species such as Porphyromonas gingivalis and Veillonella, often exceeding levels found in conventional cigarette smokers [48].

##### Mental Health Impacts

E-cigarette use has also been associated with a range of mental health concerns, including increased stress, depression, and even psychotic symptoms. Among nursing students in the Philippines, dual users of cigarettes and e-cigarettes had a twofold increased likelihood of reporting high levels of perceived stress [69]. In a United States multi-university sample, 14–15% of students who vaped in the past month reported psychotic experiences, with vaping associated with a 1.88 times higher probability of reporting such symptoms [77]. Additional studies linked e-cigarette use with a greater risk of disordered eating, inattention, and poor emotional regulation [42,50].

##### Neurobiological and Developmental Concerns

Nicotine exposure during emerging adulthood—a critical period of neurodevelopment—has been shown to disrupt reward circuitry, stress reactivity, and emotional regulation. Even single exposures have been associated with long-term alterations in affect and increased susceptibility to depressive states [42]. These neurodevelopmental disruptions may have lasting effects on cognitive and emotional functioning.

Taken together, the evidence portrays e-cigarette use among university students as a multidimensional health threat extending well beyond nicotine dependence. While addiction remains the most consistent and immediate outcome, the literature documents broader implications across respiratory, cardiovascular, oral, and mental health domains, with potential long-term neurobiological effects during a critical developmental stage. The co-occurrence of vaping with other risk behaviors further compounds vulnerability, suggesting that its impact is not isolated but embedded within wider patterns of risk-taking. Although some clinical manifestations such as EVALI are relatively rare, their severity underscores the need for preventive vigilance. Overall, the synthesis indicates that e-cigarette use in emerging adulthood entails both acute and cumulative health risks, reinforcing the importance of early, comprehensive, and multidisciplinary interventions.

#### 3.3.5. The Role of Nursing in Preventing and Managing E-Cigarette Use

Nursing plays a strategic role in the prevention, education, and management of health problems associated with e-cigarette use within university settings. From a public health perspective, this role should encompass both primary prevention—aimed at deterring the initiation of e-cigarette use among healthy young individuals—and tertiary prevention, focused on the rehabilitation of those affected by e-cigarette related diseases, including EVALI [41].

To fulfill these objectives, nurses must engage in comprehensive health education that equips university students with accurate knowledge about the composition of e-liquids, short- and long-term adverse effects, and potential neuropsychological consequences. One proposed nursing framework includes four interconnected actions: (1) assessment through detailed clinical interviews to identify individual vaping behaviors; (2) education targeting students, parents, and educators regarding e-cigarette-related risks; (3) advocacy for regulatory policies that create safer environments; and (4) research, which contributes to the evidence base and supports tailored interventions [41].

In this regard, nursing-led surveys can help assess students’ knowledge, beliefs, and behaviors related to e-cigarettes, as demonstrated in several European settings. Such tools have proven effective in identifying disparities between faculties, educational backgrounds, and levels of awareness concerning active and passive smoking [66]. Moreover, online educational programs—such as “Vaping: Know the Truth”—have been shown to significantly improve e-cigarette-related knowledge among university students, empowering them to disseminate health-promoting messages within their peer networks [45].

The nursing student community itself recognizes the importance of this role. In a study conducted in Croatia, 72% of respondents believed that nurses should play an active role in supporting e-cigarette cessation; 68% advocated for enhanced education in this area; and 52% emphasized the need for healthcare professionals to model healthy behavior by abstaining from e-cigarette use themselves [80]. These perspectives underscore the importance of integrating critical reflection, ethical responsibility, and scientific inquiry into nursing curricula [59].

Moreover, e-cigarette use has been associated with stress and anxiety, which further emphasizes the need for emotionally supportive interventions. Among university students in Southern California, more than half of JUUL users had made at least one attempt to quit—56.6% among women and 43.4% among men—reflecting a readiness for change that can be effectively addressed through behavioral strategies [67]. The SBIRT approach (Screening, Brief Intervention, and Referral to Treatment), grounded in motivational interviewing and the stages of change model, offers a promising tool for addressing chronic e-cigarette use and its associated health risks [67].

In summary, the reviewed evidence positions nursing as a cornerstone in addressing the challenges posed by e-cigarette use among university students. Nurses are uniquely situated to bridge knowledge gaps, counteract misinformation, and provide tailored interventions that encompass both individual counseling and community-level advocacy. Their involvement extends from classroom education and policy engagement to the development of culturally sensitive cessation strategies and the integration of digital health tools. Importantly, nursing students themselves recognize and demand stronger preparation in this area, underscoring the profession’s ethical responsibility to model healthy behaviors and to lead by example. Collectively, these insights highlight nursing not only as a clinical actor but as a transformative agent in shaping healthier environments and reducing the burden of e-cigarette use in emerging adulthood.

## 4. Discussion

This structured literature review provides a comprehensive synthesis of peer-reviewed evidence on e-cigarette use among university students, encompassing prevalence, patterns of use, behavioral and social determinants, knowledge and beliefs, health consequences, and implications for nursing practice. The findings reveal a high heterogeneity in prevalence rates across countries, disciplines, and demographic groups, with particularly elevated usage observed among male students, students in non-health-related programs, and users of disposable devices. E-cigarette initiation is commonly linked to the transition into university life and shaped by curiosity, peer influence, stress management, and exposure to pro-vaping content on social media. Despite widespread use, students— including those in health disciplines—demonstrate significant knowledge gaps and misconceptions, often relying on informal sources such as peers and digital platforms. Notably, many students underestimate the addictive potential and toxicological risks of e-cigarettes, and a considerable proportion view them as effective cessation tools, despite limited supporting evidence. Health consequences reported in the literature span a broad spectrum, including nicotine dependence, respiratory and cardiovascular impairments, mental health disturbances, and neurodevelopmental concerns. E-cigarette use is also associated with risk-taking behaviors and polysubstance use, reinforcing its multifaceted public health impact. Lastly, the findings of this review highlight the underexplored yet critical role of nursing in these efforts. Evidence supports the implementation of nursing-led educational initiatives, policy advocacy, and clinical interventions grounded in behavioral models. However, institutional gaps and limited curricular integration persist.

The findings of this review align with global epidemiological trends indicating a sustained increase in e-cigarette use among emerging adults. Data from the National Center for Health Statistics show that adult e-cigarette use in the United States increased from 3.7% in 2020 to 6.5% in 2023, with the highest prevalence consistently observed among individuals aged 21 to 24—an age group that largely overlaps with university student populations [82]. This demographic not only exhibits the highest rates of initiation but also a greater likelihood of regular use, reflecting both developmental vulnerability and contextual exposure during the transition to adulthood [83]. Biological sex differences remain notable, with males generally exhibiting higher usage rates, although regional and cultural variations have been reported [82,83]. Beyond biological sex, gender as a social construct that shapes roles and expectations also influence e-cigarette use. Among youth, females are more likely to report curiosity, social influence, and flavors as motives for consumption, whereas males more often cite image, peer bonding, or boredom. Nevertheless, these findings remain inconsistent and constrained by methodological heterogeneity, underscoring the need for standardized longitudinal research to clarify how gender intersects with broader sociodemographic determinants in shaping use patterns [84].

In addition to gender, disparities are evident across other sociodemographic factors. Higher prevalence has been reported among older adolescents and young adults, males, White individuals, urban residents, and those with higher educational attainment [85,86]. In some contexts, however, these patterns diverge: in Greece, higher use has been documented among women, and in Vietnam among rural residents [86]. Yet the evidence is far from uniform. Analysis of the U.S. HINTS 2017 survey showed that higher educational attainment was associated with lower e-cigarette use among White respondents but not among Black respondents, consistent with the Minorities’ Diminished Returns (MDRs) framework, which reflects structural barriers that constrain the protective effects of education in minority populations [87]. Similarly, while awareness was greater among wealthier and more educated groups, education and income did not consistently predict actual use [86]. Evidence concerning socioeconomic status, health status, occupation, or sexual orientation remains limited and inconsistent, highlighting the need to account for sociodemographic inequalities in surveillance and prevention efforts to avoid widening existing health disparities [85].

This upward trend parallels the rapid global expansion of the e-cigarette market, particularly the proliferation of disposable devices, which between 2017 and 2022 nearly tripled in nicotine strength—a phenomenon often described as the “nicotine strength arms race”—while quintupling in e-liquid capacity and dropping in price by nearly 70% [88]. From 2018 to 2022, the disposable segment grew by 116%, now accounting for nearly a quarter of the global market [89]. This growth has been fueled by the diversification of product design and the introduction of over 16,000 flavored variants—many of which are deliberately engineered to appeal to young users by masking the harshness of nicotine and other toxic compounds [90]. Crucially, marketing strategies leverage social media ecosystems, with platforms such as TikTok, Instagram, and YouTube hosting influencer-driven content that glamorizes vaping [91,92]. These channels have become powerful vectors for product promotion, bypassing traditional advertising restrictions and reinforcing e-cigarette use as a socially accepted and aesthetically appealing behavior [93].

The increasing market dominance of transnational tobacco companies adds a critical layer of complexity. Initially driven by independent manufacturers, the e-cigarette market is now heavily influenced by transnational tobacco companies. Far from promoting harm reduction, emerging evidence suggests that these companies position e-cigarettes as a complement—rather than an alternative—to traditional tobacco, aiming to preserve dual use and maintain nicotine dependence [83]. Their influence extends through aggressive digital marketing, the strategic promotion of flavor diversity, and the co-optation of harm-reduction narratives to shape policy discourse [94].

There is substantial and well-documented evidence regarding the wide array of harmful components present in electronic cigarettes, as detailed in the systematic review that informs the World Health Organization’s background report on ENDS/ENNDS [95]. Identified substances include glycols, nicotine, ultrafine particles, heavy metals, tobacco-specific nitrosamines, volatile organic compounds, hydrocarbons, polycyclic aromatic hydrocarbons, and phenols—all of which have known carcinogenic, toxic, or irritant properties [95]. Importantly, some of these compounds, such as carbonyls, have been detected even in nicotine-free e-cigarettes and are classified as potential human carcinogens and toxicants [96]. Furthermore, technical issues may occur with refillable devices, noting that improper assembly or handling may result in direct skin exposure to e-liquids during refilling processes [97]. Beyond physical health risks, emerging evidence links e-cigarette use to mental health concerns. Several studies included in this review report associations with increased stress and anxiety levels [53,59,77]. More alarmingly, a recent meta-analysis provides robust evidence of a significant association between e-cigarette use and suicidal behaviors, including ideation, planning, and attempts [98]. The study indicates a nearly 50% increase in the odds of suicidal ideation and more than a twofold increase in suicide attempts among e-cigarette users compared to non-users [98].

Despite the growing body of evidence highlighting the physical and mental health risks associated with e-cigarette use, this review reveals a persistent lack of awareness and low risk perception among university students—including those enrolled in health-related disciplines [56]. Alarmingly, some students in these programs report that they would recommend e-cigarettes to their future patients as a smoking cessation aid, despite acknowledging insufficient knowledge and a lack of preparedness to offer evidence-based guidance [43,49,63]. This misinformation not only facilitates continued use—thereby increasing harm to the users themselves—but also contributes to the normalization of behaviors such as stealth vaping in spaces explicitly designated as smoke-free.

The study by Yang et al. [99] offers a striking illustration of this phenomenon: students with more positive attitudes toward campus tobacco regulation were less likely to engage in this behavior. However, those who perceived that their peers also engaged in stealth vaping were significantly more likely to do so themselves, and more frequently. This indicates that normative beliefs may outweigh policy compliance, particularly among individuals with higher levels of e-cigarette dependence. These behavioral patterns—particularly the influence of perceived social norms on stealth vaping—are consistent with the Theory of Planned Behavior, reinforcing the importance of addressing normative beliefs in intervention design [99,100].

Beyond the regulatory implications, stealth vaping may also increase toxicant exposure for users, especially when performed repeatedly or in enclosed spaces. Crucially, it also fails to eliminate secondhand exposure for bystanders. As such, stealth vaping directly undermines the protective intent of smoke-free and aerosol-free policies on campus [101,102,103]. These findings highlight the urgent need for institutional strategies that not only strengthen enforcement mechanisms, but also correct misperceptions, improve risk communication, and foster a culture of shared responsibility for campus health.

In this context, as highlighted throughout this review, the role of nursing is pivotal in addressing misinformation and knowledge gaps through evidence-based health education initiatives [45,66]. Nurses are uniquely positioned to implement preventive strategies and deliver accurate, accessible information tailored to young adults, particularly within university settings. Public health campaigns should increasingly reflect evidence-informed priorities—such as raising awareness about the chemical constituents of e-cigarettes and their short-term health effects—which have been shown to influence cessation intentions among youth [104].

Although the effectiveness of nursing-led educational interventions in reducing e-cigarette use has been well documented—both among students and within broader academic communities—most initiatives remain concentrated in primary and secondary school environments, leaving university campuses largely unaddressed [33,35]. This gap underscores the urgent need to extend such interventions into higher education, where e-cigarette use is highly prevalent and often socially reinforced, and where health professionals in training may become future conduits of either misinformation or preventive action.

### 4.1. Strengths and Limitations

While this review offers a comprehensive and methodologically rigorous synthesis, certain limitations should be acknowledged. The absence of a quantitative meta-analysis meant that effect sizes could not be estimated, and the heterogeneity in study designs and outcomes limited direct comparability. The predominance of cross-sectional studies further constrains causal interpretation. Two eligible studies could not be fully assessed due to lack of access to the complete text—one was unavailable even through institutional library services, and another existed only as a conference abstract, as confirmed by direct author communication.

Regarding methodological quality, the appraisal using JBI and SANRA tools identified some recurrent limitations across the included studies, such as the lack of identification and control of potential confounding factors, the occasional use of non-validated measurement instruments, and, in qualitative designs, insufficient reflexivity regarding the researcher’s influence. However, these limitations were not systematic, and overall methodological quality was high: 51.2% of the studies achieved full compliance with all assessed items, and 41.9% were rated as moderate to high quality, with only one study falling below this range (62.5%). The appraisal thus served to contextualize the rigor of the included evidence and inform the interpretation of findings, rather than to diminish confidence in the synthesis.

The inclusion of diverse designs—quantitative, qualitative, longitudinal, cross-sectional, and review studies—posed challenges for direct comparison due to differences in outcome measures, analytical approaches, and depth of reporting. Nonetheless, this diversity also constitutes a strength, as it enabled an integrative synthesis that captures both measurable patterns and the contextual, behavioral, and social dimensions of e-cigarette use among university students. Careful and systematic integration ensured that the synthesis remained as faithful as possible to the original data while providing a global perspective.

Lastly, the geographical distribution of studies was uneven, with a predominance of research from the United States, parts of Europe, the Middle East, and Asia, but limited representation from Eastern Europe, Oceania (except New Zealand), Latin America, and no representation from Sub-Saharan Africa. The restriction to articles published in English or Spanish may also have introduced language bias. These factors may limit the applicability of the findings to underrepresented regions and populations.

Several strengths mitigate the potential impact of these limitations. The structured narrative approach, guided by SANRA criteria, enhanced transparency and replicability, while accommodating a wide variety of study designs. The use of the PICO framework provided conceptual clarity and strengthened the alignment between the search strategy and thematic synthesis. The application of design-specific JBI appraisal tools ensured a robust evaluation of methodological rigor, and the inclusion of studies from multiple countries and disciplines enhances the contextual relevance and external validity of the findings. Taken together, these limitations and strengths may have influenced the completeness of the evidence base (internal validity) and could modestly restrict the generalizability of the findings to cultural and regulatory contexts not represented in the included studies (external validity). While certain methodological shortcomings were identified, the overall quality of the evidence was high, and the structured synthesis mitigated many of these limitations. Therefore, the conclusions of this review remain robust, though they should be interpreted with consideration of the specific geographical and methodological scope of the included research.

### 4.2. Main Findings and Their Influence on Further Interventions

This review identifies several critical patterns with direct implications for prevention and intervention strategies in university settings. First, e-cigarette initiation often occurs during the transition into university life, driven by curiosity, peer influence, stress management, and exposure to pro-vaping digital content. These findings suggest that interventions must address both social and digital environments, targeting early university stages to prevent initiation

Second, despite widespread use, including among health sciences students, knowledge gaps and misconceptions persist regarding the chemical composition, addictive potential, and health risks of e-cigarettes. Misperceptions about their effectiveness as smoking cessation tools are common, and many students rely on informal sources for information. This underscores the need for credible, accessible, and discipline-specific health education that corrects misinformation and fosters critical appraisal of marketing narratives.

Third, the evidence links e-cigarette uses to a wide range of adverse outcomes—nicotine dependence, respiratory and cardiovascular impairment, oral health alterations, mental health disturbances, and neurodevelopmental risks—as well as to polysubstance use and other risk-taking behaviors. These findings highlight the importance of integrating cessation support within broader risk-reduction frameworks that also address co-occurring behaviors.

Lastly, the review reveals institutional and policy gaps, including limited curricular integration of vaping-related content and insufficient enforcement of smoke-free campus regulations. These gaps undermine prevention efforts and create opportunities for behaviors such as stealth vaping, which both perpetuate nicotine dependence and increase secondhand exposure.

These findings collectively inform the targeted recommendations outlined in the next section.

### 4.3. Recommendations for the Future

From a practical standpoint, universities should prioritize the development and implementation of structured, evidence-based health education campaigns—ideally led by nursing professionals—that explicitly address the chemical composition, addictive potential, and short-term health consequences of e-cigarette use. These interventions should be culturally and developmentally tailored, leveraging digital platforms and peer education models to better engagement.

Greater efforts are needed to integrate vaping-related content into health sciences curricula, ensuring that future healthcare providers are adequately trained to deliver cessation guidance rooted in current evidence. Institutional policies must also move beyond symbolic declarations and adopt concrete enforcement mechanisms, accompanied by clear communication strategies, cessation support services, and dedicated health promotion resources.

Future research should evaluate the effectiveness of university-based interventions, particularly those grounded in behavioral theories such as the Theory of Planned Behavior. Longitudinal studies are warranted to examine causal pathways linking e-cigarette use to psychosocial, academic, and health-related outcomes, as well as to explore the mediating role of digital exposure and normative beliefs. Moreover, qualitative investigations could deepen our understanding of user experiences, motivations, and barriers to cessation—especially among high-risk subgroups such as dual users or individuals with underlying mental health conditions. Comparative studies across countries and academic disciplines would also offer valuable insights into regulatory environments, social norms, and educational exposure shape usage patterns. Taken together, these lines of inquiry are essential to inform comprehensive, interdisciplinary responses that mitigate the rising burden of e-cigarette use among university students.

## 5. Conclusions

This review highlights the widespread use of e-cigarettes among university students and the persistence of misinformation, low risk perception, and normalization of use—particularly in the context of stealth vaping. Despite increasing evidence of physical and mental health risks, misconceptions remain even among health sciences students, underscoring the urgent need for targeted education.

Nursing professionals play a key role in addressing these gaps through health education, policy advocacy, and campus-based interventions. Future efforts should prioritize evidence-informed strategies tailored to university settings, and further research is needed to explore long-term health outcomes and the effectiveness of prevention initiatives in higher education.

## Data Availability

The data presented in this study are available upon reasonable request. The data are available from María-Ángeles Núñez-Baila (email: mnbaila@us.es).

## Supplementary Materials

The following supporting information can be downloaded at: https://www.mdpi.com/article/10.3390/healthcare13172150/s1, Supplementary Material S1. Methodological Quality Assessment Checklists. Supplementary Material S2. Summary of Methodological Quality Assessment of Included Studies According to Study Design and Appraisal Tool (JBI or SANRA). References [105,106,107,108,109,110,111] are included in the Supplementary Material.

## Abbreviations

The following abbreviations are used in this manuscript:

CBD	Cannabiol
EVALI	E-cigarette or vaping product use-associated lung
JBI	Joanna Briggs Institute
SANRA	The Assessment of Narrative Review Articles
SBIRT	Screening, Brief Intervention, and Referral to Treatment.
THC	Tetrahydrocannabinol

## References

[1] World Health Organization Tobacco. https://www.who.int/health-topics/tobacco.

[2] Robayo-González C.X., Becerra N., Castro-Goyes D.F. (2019). Effects of electronic cigarettes on health. A literature review. Rev. Salud Pública.

[3] Tarrazo M., Pérez-Ríos M., Santiago-Pérez M.I., Malvar A., Suanzes J., Hervada X. (2017). Changes in tobacco consumption: Boom of roll-your-own cigarettes and emergence of e-cigarettes. Gac. Sanit..

[4] Lidón-Moyano C., Martínez-Sánchez J.M., Fu M., Ballbè M., Martín-Sánchez J.C., Fernández E. (2016). Prevalence and user profile of electronic cigarettes in Spain (2014). Gac. Sanit..

[5] Soneji S., Barrington-Trimis J.L., Wills T.A., Leventhal A.M., Unger J.B., Gibson L.A., Yang J., Primack B.A., Andrews J.A., Miech R.A. (2017). Association between initial use of e-cigarettes and subsequent cigarette smoking among adolescents and young adults: A systematic review and meta-analysis. JAMA Pediatr..

[6] Brożek G.M., Jankowski M., Lawson J.A., Shpakou A., Poznański M., Zielonka T.M., Klimatckaia L., Loginovich Y., Rachel M., Gereová J. (2019). The prevalence of cigarette and e-cigarette smoking among students in Central and Eastern Europe—Results of the YUPESS Study. Int. J. Environ. Res. Public Health.

[7] Farsalinos K.E., Poulas K., Voudris V., Le Houezec J. (2016). Electronic cigarette use in the European Union: Analysis of a representative sample of 27 460 Europeans from 28 countries. Addiction.

[8] Jackson S.E., Tattan-Birch H., Shahab L., Brown J. (2024). Trends in long term vaping among adults in England, 2013–2023: Population based study. BMJ.

[9] Lyzwinski L.N., Naslund J.A., Miller C.J., Eisenberg M.J. (2022). Global youth vaping and respiratory health: Epidemiology, interventions, and policies. NPJ Prim. Care Respir. Med..

[10] Marques P., Piqueras L., Sanz M.-J. (2021). An updated overview of e-cigarette impact on human health. Respir. Res..

[11] Kopa-Stojak P.N., Pawliczak R. (2025). Disposable electronic cigarettes–chemical composition and health effects of their use. A systematic review. Toxicol. Mech. Methods.

[12] Cheng T. (2014). Chemical evaluation of electronic cigarettes. Tob. Control.

[13] Chun L.F., Moazed F., Calfee C.S., Matthay M.A., Gotts J.E. (2017). Pulmonary toxicity of e-cigarettes. Am. J. Physiol. Lung Cell. Mol. Physiol..

[14] Hadwiger M.E., Trehy M.L., Ye W., Moore T., Allgire J., Westenberger B. (2010). Identification of amino-tadalafil and rimonabant in electronic cigarette products using high pressure liquid chromatography with diode array and tandem mass spectrometric detection. J. Chromatogr. A.

[15] Thirión-Romero I., Pérez-Padilla R., Zabert G., Barrientos-Gutiérrez I. (2019). Respiratory impact of electronic cigarettes and “low risk” tobacco. Rev. Investig. Clin..

[16] Leventhal A.M., Strong D.R., Kirkpatrick M.G., Unger J.B., Sussman S., Riggs N.R., Stone M.D., Khoddam R., Samet J.M., Audrain-McGovern J. (2015). Association of electronic cigarette use with initiation of combustible tobacco product smoking in early adolescence. JAMA.

[17] Klager S., Vallarino J., MacNaughton P., Christiani D.C., Lu Q., Allen J.G. (2017). Flavoring chemicals and aldehydes in e-cigarette emissions. Environ. Sci. Technol..

[18] Christiani D.C. (2020). Vaping-induced acute lung injury. N. Engl. J. Med..

[19] O’Callaghan M., Boyle N., Fabre A., Keane M.P., McCarthy C. (2022). Vaping-associated lung injury: A review. Medicina.

[20] Zulfiqar H., Sankari A., Rahman O. (2025). Vaping-associated pulmonary injury. StatPearls.

[21] Jonas A.M., Raj R. (2020). Vaping-related acute parenchymal lung injury: A systematic review. Chest.

[22] Malani P.N., Walter K.L. (2024). What are e-cigarettes?. JAMA.

[23] Breland A., Soule E., Lopez A., Ramôa C., El-Hellani A., Eissenberg T. (2017). Electronic cigarettes: What are they and what do they do?. Ann. New York Acad. Sci..

[24] Razali M.F., Leong Y.-H., Mohd Samin A.S. (2025). History and Evolution of E-Cigarettes. E-Cigarettes: Risks, Research and Challenges.

[25] Mariana D. (2023). Harmful effects of electronic cigarette on human health. A review. Rev. Colomb. Neumol..

[26] Worl Health Organization (2024). WHO Report on the Global Tobacco Epidemic, 2025: Warning About the Dangers of Tobacco.

[27] Arnett J.J. (2024). Emerging adulthood: The winding road from the late teens through the twenties. Emerging Adulthood: The Winding Road from the Late Teens Through the Twenties.

[28] Arnett J.J., Žukauskienė R., Sugimura K. (2014). The new life stage of emerging adulthood at ages 18–29 years: Implications for mental health. Lancet Psychiatry.

[29] Allem J.-P., Forster M., Neiberger A., Unger J.B. (2015). Characteristics of emerging adulthood and e-cigarette use: Findings from a pilot study. Addict. Behav..

[30] Geindreau D., Girault A., Gallopel-Morvan K. (2024). Tobacco-free university campus policies: A systematic review. J. Am. Coll. Health.

[31] Russell A.M., Yang M., Barry A.E., Merianos A.L., Lin H.-C. (2022). Stealth vaping among college students on four geographically distinct tobacco-free college campuses: Prevalence and practices. Nicotine Tob. Res..

[32] Gholap V.V., Kosmider L., Golshahi L., Halquist M.S. (2020). Nicotine forms: Why and how do they matter in nicotine delivery from electronic cigarettes?. Expert. Opin. Drug Deliv..

[33] Smith L.M., Boehm L., Strang L.V., DeMarre C., Marcyjanik D. (2024). Targeted education for school staff on electronic nicotine delivery systems: A Nurse Led Intervention. J. Sch. Nurs..

[34] McGee P.L., Goldschmidt K. (2019). E-Cigarettes and Vaping: What do pediatric nurses need to know?. J. Pediatr. Nurs..

[35] Russell A.J., Shishani K., Hurst S. (2025). The role of the school nurse in e-cigarette prevention and cessation: A scoping review. J. Sch. Nurs..

[36] Gardner L.A., Rowe A.-L., Newton N.C., Egan L., Hunter E., Devine E.K., Aitken T., Thornton L., Teesson M., Stockings E. (2024). A systematic review and meta-analysis of school-based preventive interventions targeting e-cigarette use among adolescents. Prev. Sci..

[37] Patil S., Fageeh H.N., Mushtaq S., Ajmal M., Chalikkandy S.N., Ashi H., Ahmad Z.H., Khan S.S., Khanagar S., Varadarajan S. (2022). Prevalence of electronic cigarette usage among medical students in Saudi Arabia—A systematic review. Niger. J. Clin. Pract..

[38] Baethge C., Goldbeck-Wood S., Mertens S. (2019). SANRA—A Scale for the Quality assessment of narrative review articles. Res. Integr. Peer Rev..

[39] Dobbs P.D., Clawson A.H., Gowin M., Cheney M.K. (2020). Where college students look for vaping information and what information they believe. J. Am. Coll. Health.

[40] Fernández-García D., Ordás B., Fernández-Peña R., Bárcena-Calvo C., Ordoñez C., Amo-Setién F.J., Gómez-Salgado J., Martínez-Isasi S. (2020). Smoking in nursing students: A prevalence multicenter study. Medicine.

[41] Huey S., Tierney C., Granitto M., Brien L. (2020). The vaping epidemic: Calling nurses to action. Nursing.

[42] Jun J., Kim J. (2021). How do colleges communicate about e-cigarettes? The presentation of risk, policy, and cessation resources on college websites. J. Am. Coll. Health.

[43] Natto Z.S. (2020). Dental students’ knowledge and attitudes about electronic cigarettes: A cross-sectional study at one Saudi university. J. Dent. Educ..

[44] Prigitano A., Binda S., Pariani E. (2020). Tobacco and e-cigarette smoking habits among Italian healthcare students. Ann. Ig..

[45] Pulvers K., Correa J.B., Krebs P., El Shahawy O., Marez C., Doran N., Myers M. (2021). JUUL e-cigarette quit attempts and cessation perceptions in college student JUUL e-cigarette users. Am. J. Health Promot..

[46] Wamamili B., Wallace-Bell M., Richardson A., Grace R.C., Coope P. (2020). Electronic cigarette use among university students aged 18–24 years in New Zealand: Results of a 2018 national cross-sectional survey. BMJ Open.

[47] Wang W., Lu M., Cai Y., Feng N. (2020). Awareness and use of e-cigarettes among university students in Shanghai, China. Tob. Induc. Dis..

[48] Almeida-da-Silva C.L.C., Matshik Dakafay H., O’Brien K., Montierth D., Xiao N., Ojcius D.M. (2021). Effects of electronic cigarette aerosol exposure on oral and systemic health. Biomed. J..

[49] Alzahrani S., Alghamdi R.A., Almutairi A.M., Alghamdi A.A., Aljuhani A.A., ALbalawi A.H. (2021). Knowledge and attitudes among medical students toward the clinical usage of e-cigarettes: A cross-sectional study in a University Hospital in Saudi Arabia. Risk Manag. Healthc. Policy.

[50] Ganson K.T., Nagata J.M. (2021). Associations between vaping and eating disorder diagnosis and risk among college students. Eat. Behav..

[51] Jones R.D., Asare M., Lanning B. (2021). A retrospective cross-sectional study on the prevalence of e-cigarette use among college students. J. Community Health.

[52] Kurdi R., Al-Jayyousi G.F., Yaseen M., Ali A., Mosleh N., Abdul Rahim H.F. (2021). Prevalence, risk factors, harm perception, and attitudes toward e-cigarette use among university students in Qatar: A cross-sectional study. Front. Public Health.

[53] Newcombe K.V., Dobbs P.D., Oehlers J.S., Dunlap C.M., Cheney M.K. (2021). College students’ reasons for using JUULs. Am. J. Health Promot..

[54] Omoike O.E., Johnson K.R. (2021). Prevalence of vaping and behavioral associations of vaping among a community of college students in the United States. J. Community Health.

[55] Oh H., Banawa R., Lee J.O., Zhou S., Huh J. (2021). Vaping and psychotic experiences among college students in the United States. Drug Alcohol Depend..

[56] Páez C.S., Orellana H.D.I., Nazzal N.C.P. (2021). Electronic cigarettes perception and prevalence among medical students. Rev. Chil. Enferm. Respir..

[57] Phetphum C., Prajongjeep A., Thawatchaijareonying K., Wongwuttiyan T., Wongjamnong M., Yossuwan S., Surapon D. (2021). Personal and perceptual factors associated with the use of electronic cigarettes among university students in Northern Thailand. Tob. Induc. Dis..

[58] Pougnet R., Chapalain D., Fortin C., Loddé B., Eniafe-Eveilard B.M., Pougnet L., Dewitte J.D., Le Denmat V. (2021). Cigarette and e-cigarette use among a sample of French health students. Rev. Mal. Respir..

[59] Rayman L., Kessler T.A. (2021). E-cigarettes in college-age students: Roles of the nurse practitioner. JNP.

[60] Tarran R., Barr R.G., Benowitz N.L., Bhatnagar A., Chu H.W., Dalton P., Doerschuk C.M., Drummond M.B., Gold D.R., Goniewicz M.L. (2021). E-Cigarettes and cardiopulmonary health. Function.

[61] Worthen M., Ahmad I. (2023). Participatory research on the prevalence of multi-substance vaping in college students. J. Am. Coll. Health.

[62] AlMuhaissen S., Mohammad H., Dabobash A., Nada M.Q., Suleiman Z.M. (2022). Prevalence, knowledge, and attitudes among health professions students toward the use of electronic cigarettes. Healthcare.

[63] Babjaková J., Rimárová K., Weitzman M., Bušová M., Jurkovičová J., Dorko E., Argalášová Ľ. (2022). E-Cigarette use, opinion about harmfulness and addiction among university students in Bratislava, Slovakia. Cent. Eur. J. Public Health.

[64] McLeish A.C., Hart J.L., Walker K.L. (2022). College student e-cigarette users’ knowledge about e-cigarettes: Ingredients, health risks, device modifications, and information sources. Int. J. Environ. Res. Public Health.

[65] Seidel A.-K., Morgenstern M., Galimov A., Pedersen A., Isensee B., Goecke M., Hanewinkel R. (2022). Use of electronic cigarettes as a predictor of cannabis experimentation: A longitudinal study among German youth. Nicotine Tob. Res..

[66] Hair E.C., Tulsiani S., Aseltine M., Do E.K., Lien R., Zapp D., Green M., Vallone D. (2024). Vaping—Know the truth: Evaluation of an online vaping prevention curriculum. Health Promot. Pract..

[67] Holden J., Simerson D. (2023). Screening, Brief Intervention and Referral to Treatment (SBIRT) by nurses to college students who use electronic cigarettes. J. Am. Coll. Health.

[68] Kaewsutha N., Karawekpanyawong R. (2023). Tobacco and e-cigarette use among Thai dental students: A cross-sectional national survey, 2021. J. Int. Soc. Prev. Community Dent..

[69] Resano J.E.P., Regencia Z.J.G., Baja E.S. (2023). Association between smoking and vaping usage and perceived stress levels of undergraduate nursing students in Manila, Philippines. J. Public Health Emerg..

[70] Vilcassim M.J.R., Jacob D., Stowe S., Fifolt M., Zierold K.M. (2023). Sex differences in electronic cigarette device use among college students. J. Community Health.

[71] Albadrani M.S., Tobaiqi M.A., Muaddi M.A., Eltahir H.M., Abdoh E.S., Aljohani A.M., Albadawi E.A., Al-zaman N.S., Abouzied M.M., Fadlalmola H.A. (2024). A global prevalence of Electronic Nicotine Delivery Systems (ENDS) use among students: A systematic review and meta-analysis of 4,189,145 Subjects. BMC Public Health.

[72] Bataineh B.S., Hébert E.T., Loukas A., Harrell M.B., Yang Q., Murthy D., Schwartz S., Jung S., Wilkinson A.V. (2024). Problematic social media use and vaping among Mexican-American college students. Digit. Health.

[73] Folivi F., Petrey A.M., Bravo A.J., Holt L.J., Looby A. (2025). Stimulant Norms and Prevalence (SNAP) Study Team Need frustration and e-cigarette use and dependence among college students: The mediating role of ruminative thinking. Subst. Use Misuse.

[74] Kinouani S., Da Cruz H., Simon M., Abraham M., Perret G., Langlois E., Tzourio C. (2025). The transition from cigarette smoking to the exclusive or partial use of e-cigarettes: A multi-stage mixed methods study among French university students. Addict. Behavs..

[75] Maqsood A., Shahidan W.N.S., Mirza D., Ahmed N., Heboyan A. (2024). Social acceptability and health concerns of smoking and vaping among university students: A cross-sectional study. Tob. Use Insights.

[76] Mostafa O.A., Taha M.A. (2024). Knowledge, attitude, and use of electronic cigarettes among Cairo university medical students. J. Egypt. Public Health Assoc..

[77] Roh T., Fields S., Sahu R., Trisha N.F., Carrillo G. (2024). Vaping behavior and intention to quit among undergraduate students in a Hispanic-serving university. J. Community Health.

[78] Singer J.M., Tackett A.P., Alalwan M.A., Roberts M.E. (2024). Nicotine dependence among undergraduates who use nicotine salt-based e-cigarettes. J. Am. Coll. Health.

[79] Ou T.-S., Buu A., Yang J.J., Lin H.-C. (2024). E-cigarette use reasons and associated e-cigarette use dependence among college students: A longitudinal examination. Addict. Behav..

[80] Kajan L., Puljak L., Matić I., Marendić M., Zoranić S., Ivanišević K., Majstorović D., Puharić Z., Skitarelić N., Neuberg M. (2025). Usage, knowledge and attitudes towards electronic cigarettes use among nursing students in Croatia: A cross-sectional study. BMC Nurs..

[81] Soerianto W., Jaspers I. (2025). E-cigarette, or vaping, product use associated lung injury: Epidemiology, challenges, and implications with COVID-19. Pediatr. Pulmonol..

[82] Valhratian A., Briones E., Jamal A., Marynak K., Valenzuela C. Electronic Cigarette Use Among Adults in the United States; NCHS Data Briefs, National Center for Health Statistics (U.S.), 30 January 2025; National Health Interview Survey. https://stacks.cdc.gov/view/cdc/174583.

[83] World Health Organization (2016). Electronic Nicotine Delivery Systems and Electronic Non-Nicotine Delivery Systems (ENDS/ENNDS).

[84] Bedi M.K., Bedi D.K., Ledgerwood D.M. (2022). Gender differences in reasons for using electronic cigarettes: A systematic review. Nicotine Tob. Res..

[85] Hartwell G., Thomas S., Egan M., Gilmore A., Petticrew M. (2017). E-Cigarettes and Equity: A systematic review of differences in awareness and use between sociodemographic groups. Tob. Control.

[86] Kundu S., Shaw S., Khan J., Chattopadhyay A., Baptista E.A., Paswan B. (2023). Age, Gender and socioeconomic patterns of awareness and usage of e-cigarettes across selected WHO region countries: Evidence from the global adult tobacco survey. BMJ Open.

[87] Assari S., Mistry R., Bazargan M. (2020). Race, Educational Attainment, and E-Cigarette Use. J. Med. Res. Innov..

[88] Diaz M.C., Silver N.A., Bertrand A., Schillo B.A. (2025). Bigger, Stronger and Cheaper: Growth in e-cigarette market driven by disposable devices with more e-liquid, higher nicotine concentration and declining prices. Tob. Control.

[89] Plesa S. Disposable E-Cigarettes: Global Market Overview, May 2022. ECigIntelligence 2022. https://ecigintelligence.com/content_types/market-reports/?geography=south-america.

[90] Hsu G., Sun J.Y., Zhu S.-H. (2018). Evolution of electronic cigarette brands from 2013–2014 to 2016–2017: Analysis of brand websites. J. Med. Internet Res..

[91] Jung S., Murthy D., Bateineh B.S., Loukas A., Wilkinson A.V. (2024). The normalization of vaping on TikTok using computer vision, natural language processing, and qualitative thematic analysis: Mixed methods study. J. Med. Internet Res..

[92] Silver N.A., Bertrand A., Kucherlapaty P., Schillo B.A. (2023). Examining influencer compliance with advertising regulations in branded vaping content on Instagram. Front. Public Health.

[93] Ramamurthi D., Fadadu R.P., Jackler R.K. (2016). Electronic cigarette marketers manipulate antitobacco advertisements to promote vaping. Tob. Control.

[94] World Health Organization (2023). Electronic Cigarettes: Call to Action.

[95] Pisinger C., Døssing M. (2014). A systematic review of health effects of electronic cigarettes. Prev. Med..

[96] Kosmider L., Sobczak A., Fik M., Knysak J., Zaciera M., Kurek J., Goniewicz M.L. (2014). Carbonyl compounds in electronic cigarette vapors: Effects of nicotine solvent and battery output voltage. Nicotine Tob. Res..

[97] Lee J., Kong G., Krishnan-Sarin S., Camenga D.R. (2024). Unintended exposure to e-liquids and subsequent health outcomes among US youth and dults. PLoS ONE.

[98] Awad A.A., Itumalla R., Gaidhane A.M., Khatib M.N., Ballal S., Bansal P., Srivastava M., Arora I., Kumar M.R., Sinha A. (2024). Association of electronic cigarette use and suicidal behaviors: A systematic review and meta-analysis. BMC Psychiatry.

[99] Yang M., Russell A.M., Barry A.E., Merianos A.L., Lin H.-C. (2023). Stealth vaping and associated attitudes, perceptions, and control beliefs among US college students across four tobacco-free campuses. Addict. Behav..

[100] Hagger M.S., Hamilton K. (2025). Progress on Theory of Planned Behavior Research: Advances in research synthesis and agenda for future research. J. Behav. Med..

[101] Su W.-C., Lin Y.-H., Wong S.-W., Chen J.Y., Lee J., Buu A. (2021). Estimation of the dose of electronic cigarette chemicals deposited in human airways through passive vaping. J. Expo. Sci. Environ. Epidemiol..

[102] Su W.-C., Wong S.-W., Buu A. (2021). Deposition of e-cigarette aerosol in human airways through passive vaping. Indoor Air.

[103] Cherian S.V., Kumar A., Estrada-Y-Martin R.M. (2020). E-Cigarette or Vaping Product-Associated Lung Injury: A Review. Am. J. Med..

[104] Eggers M.E., Fajobi O., Kelly L.K., Thompson J., Nonnemaker J.M. (2023). Young adult–targeted vaping cessation media campaigns: Promising themes. Health Educ..

[105] Moola S., Munn Z., Tufanaru C., Aromataris E., Sears K., Sfetcu R., Currie M., Qureshi R., Mattis P., Lisy K., Aromataris E., Munn Z. (2020). Chapter 7: Systematic reviews of etiology and risk. JBI Manual for Evidence Synthesis.

[106] Lockwood C., Munn Z., Porritt K. (2015). Qualitative research synthesis: Methodological guidance for systematic reviewers utilizing meta-aggregation. Int. J. Evid. Based Healthc..

[107] Munn Z., Moola S., Lisy K., Riitano D., Tufanaru C. (2015). Methodological guidance for systematic reviews of observational epidemiological studies reporting prevalence and incidence data. Int. J. Evid. Based Healthc..

[108] Naing L., Winn T., Rusli B.N. (2006). Practical issues in calculating the sample size for prevalence studies. Arch. Orofac. Sci..

[109] Daniel W.W. (1999). Biostatistics: A Foundation for Analysis in the Health Sciences.

[110] Aromataris E., Fernandez R., Godfrey C., Holly C., Kahlil H., Tungpunkom P. (2015). Summarizing systematic reviews: Methodological development, conduct and reporting of an Umbrella review approach. Int. J. Evid. Based Healthc..

[111] Whiting P., Rutjes A.W.S., Reitsma J.B., Bossuyt P.M.M., Kleijnen J. (2003). The development of QUADAS: A tool for the quality assessment of studies of diagnostic accuracy included in systematic reviews. BMC Med. Res. Methodol..

