# Supplementary material for: E-Cigarette Use Among University Students: A Structured Literature Review of Health Risks, Behavioral and Social Determinants, and Nursing Implications
Source: Healthcare (Basel). 2025 Aug 28;13(17):2150. doi: 10.3390/healthcare13172150 (PMC12428087; doi:10.3390/healthcare13172150)
Supplement: Supplementary file 1 [file healthcare-13-02150-s001.zip › healthcare-3814389-supplementary.pdf]

Supplementary Material S1. Methodological Quality Assessment Checklists

Scale for the Assessment of Narrative Review Articles – SANRA [38]

Please rate the quality of the narrative review article in quest ion, using categories 0–2 on the following scale. For each aspect of quality, please choose the option which best fits your evaluation, using categories 0 and 2 freely to imply general low and high quality.

These are not intended to imply the worst or best imaginable quality.

1) Justification of the article' s importance for the readership

- The importance is not justified. 0
- The importance is alluded to, but not explicitly justified. 1
- The importance is explicitly justified. 2

2) Statement of concrete aims or formulation of questions

- No aims or questions are formulated. 0
- Aims are formulated generally but not concretely or in terms of clear questions. 1
- One or more concrete aims or questions are formulated. 2

3) Description of th e literature search

- The search strategy is not presented. 0
- The literature search is described briefly. 1
- The literature search is described in detail, including search terms and inclusion criteria. 2

4) Referencing

- Key statements are not supported by references. 0
- The referencing of key statements is inconsistent. 1
- Key statements are supported by references. 2

5) Scientific reasoning (e.g., incorporation of appropriate evidence, such as RCTs in clinical medicine)

- The article's point is not based on appropriate arguments. 0
- Appropriate evidence is introduced selectively. 1
- Appropriate evidence is generally present. 2

6) Appropriate presentation of data (e.g., absolute vs relative risk; effect sizes without confidence intervals)

- Data are presented inadequately. 0
- Data are often not presented in the most appropriate way. 1
- Relevant outcome data are generally presented appropriately. 2

Sumscore:

SANRA – explanations and instructions

This scale is intended to help editors assess the quality of a narrative review article based on formal criteria accessible to the reader.

It cannot cover other elements of editorial decision making such as degree of originality, topicality, conflicts of interest or the plausibility, correctness or completeness of the content itself. SANRA is an instrument for editors, authors, and reviewers evaluating individual

manuscripts. It may also help editors to document average manuscript quality within their journal and researchers to document the manuscript quality, for example in peer review research. Using only three scoring options, 0, 1 and 2, SANRA is intended to provide a swift and pragmatic sum score for quality, for everyday use with real manuscripts, in a field where established quality standards have previously been lacking. It is not designed as an exact measurement of the quality of all theoretically possible manuscripts. For this reason, the extreme values (0 and 2) should be used relatively freely and not reserved only for perfect or hopeless articles.

We recommend that users test-rate a few manuscripts to familiarize themselves with the scale, before using it on the intended group of manuscripts. Ratings should assess the totality of a manuscript, including the abstract. The following comments clarify how each question is designed to be used.

Item 1 – Justification of the article's importance for the readership

Justification of importance for the readership must be seen in the context of each journal's readership. Consider how well the manuscript outlines the clinical problem and highlights unanswered questions or evidence gaps – thoroughly (2), superficially (1), or not at all (0).

Item 2 – Statement of concrete/specific aims or formulation of questions

A good paper will propose one or more specific aims or questions which will be dealt with or topics which will be reviewed. Please rate whether this has been done thoroughly and clearly (2), vaguely or unclearly (1), or not at all (0).

Item 3 – Description of the literature search

A convincing narrative review will be transparent about the sources of information on which the text is based. Please rate the degree to which you think this has been achieved. To achieve a rating of 2, it is not necessary to describe the literature search in as much detail as for a systematic review (searching multiple databases, including exact descriptions of search history, flowcharts, etc.), but it is necessary to specify search terms, and the types of literature included. A manuscript which only refers briefly to its literature search would score 1, while one not mentioning its methods would score 0.

Item 4 – Referencing

No manuscript references all statements. However, those that are essential for the arguments of the manuscript – "key statements" – should be backed by references in all or almost all cases. Exceptions could reasonably be made for rating purposes where a key statement has uncontroversial face-validity, such as "Diabetes is among the commonest causes of chronic morbidity worldwide." Please rate the completeness of referencing: for most or all relevant key statements (2), inconsistently (1), sporadically (0).

Item 5 – Scientific reasoning

The item describes the quality of the scientific point made. A convincing narrative review presents evidence for key arguments. It should mention study design (randomized controlled trial, qualitative study, etc.), and where available levels of evidence. Please rate whether you feel this has been done thoroughly (2), superficially (1), or hardly at all (0). Unlike item 6, which is concerned with the selection and presentation of concrete outcome data, this item relates to the use of evidence and of types of evidence in the manuscript's arguments.

Item 6 – Appropriate presentation of data:

This item describes the correct presentation of data central to the article's argument. Which data are considered relevant varies from field to field. In some areas relevant data would be absolute rather than relative risks or clinical versus surrogate or intermediate endpoints. These outcomes must be presented correctly. For example, it is appropriate that effect sizes are accompanied by confidence intervals. Please rate how far the paper achieves this – thoroughgoingly (2), partially (1), or hardly at all (0). Unlike item 5, which relates to the use of evidence and of types of evidence in the manuscript's arguments, this item is concerned with the selection and presentation of concrete outcome data.

JBI CRITICAL APPRAISAL CHECKLIST FOR ANALYTICAL CROSS SECTIONAL STUDIES

Reviewer_______________________________________Date_______________________________

Author____Year_________ Record Number_________

	Yes	No	Unclear	Not applicable
1. Were the criteria for inclusion in the sample clearly defined?	□	□	□	□
2. Were the study subjects and the setting described in detail?	□	□	□	□
3. Was the exposure measured in a valid and reliable way?	□	□	□	□
4. Were objective, standard criteria used for measurement of the condition?	□	□	□	□
5. Were confounding factors identified?	□	□	□	□
6. Were strategies to deal with confounding factors stated?	□	□	□	□
7. Were the outcomes measured in a valid and reliable way?	□	□	□	□
8. Was appropriate statistical analysis used?	□	□	□	□

Overall appraisal: Include □ Exclude □ Seek further info □

Comments (Including reason for exclusion)

____

____

____

Critical Appraisal Checklist for Analytical Cross Sectional Studies

© JBI, 2020. All rights reserved. JBI grants use of these tools for research purposes only. All other enquiries should be sent to jbisynthesis@adelaide.edu.au.

EXPLANATION OF ANALYTICAL CROSS SECTIONAL STUDIES CRITICAL APPRAISAL [105]

Analytical cross sectional studies Critical Appraisal Tool

Answers: Yes, No, Unclear or Not/Applicable

1. Were the criteria for inclusion in the sample clearly defined?

The authors should provide clear inclusion and exclusion criteria that they developed prior to recruitment of the study participants. The inclusion/exclusion criteria should be specified (e.g., risk, stage of disease progression) with sufficient detail and all the necessary information critical to the study.

2. Were the study subjects and the setting described in detail?

The study sample should be described in sufficient detail so that other researchers can determine if it is comparable to the population of interest to them. The authors should provide a clear description of the population from which the study participants were selected or recruited, including demographics, location, and time period.

3. Was the exposure measured in a valid and reliable way?

The study should clearly describe the method of measurement of exposure. Assessing validity requires that a 'gold standard' is available to which the measure can be compared. The validity of exposure measurement usually relates to whether a current measure is appropriate or whether a measure of past exposure is needed.

Reliability refers to the processes included in an epidemiological study to check repeatability of measurements of the exposures. These usually include intra-observer reliability and inter-observer reliability.

4. Were objective, standard criteria used for measurement of the condition?

It is useful to determine if patients were included in the study based on either a specified diagnosis or definition. This is more likely to decrease the risk of bias. Characteristics are another useful approach to matching groups, and studies that did not use specified diagnostic methods or definitions should provide evidence on matching by key characteristics

5. Were confounding factors identified?

Confounding has occurred where the estimated intervention exposure effect is biased by the presence of some difference between the comparison groups (apart from the exposure investigated/of interest). Typical confounders include baseline characteristics, prognostic factors, or concomitant exposures (e.g. smoking). A confounder is a difference between the comparison groups and it influences the direction of the study results. A high quality study at the level of cohort design will identify the potential confounders and measure them (where possible). This is difficult for studies where behavioral, attitudinal or lifestyle factors may impact on the results.

6. Were strategies to deal with confounding factors stated?

Strategies to deal with effects of confounding factors may be dealt within the study design or in data analysis. By matching or stratifying sampling of participants, effects of confounding factors can be adjusted for. When dealing with adjustment in data analysis, assess the statistics used in the study. Most will be some form of multivariate regression analysis to account for the confounding factors measured.

7. Were the outcomes measured in a valid and reliable way?

Read the methods section of the paper. If for e.g. lung cancer is assessed based on existing definitions or diagnostic criteria, then the answer to this question is likely to be yes. If lung cancer is assessed using observer reported, or self-reported scales, the risk of over- or under-reporting is increased, and objectivity is compromised. Importantly, determine if the measurement tools used were validated instruments as this has a significant impact on outcome assessment validity.

Having established the objectivity of the outcome measurement (e.g. lung cancer) instrument, it's important to establish how the measurement was conducted. Were those involved in collecting data trained or educated in the use of the instrument/s? (e.g. radiographers). If there was more than one data collector, were they similar in terms of level of education, clinical or research experience, or level of responsibility in the piece of research being appraised?

8. Was appropriate statistical analysis used?

As with any consideration of statistical analysis, consideration should be given to whether there was a more appropriate alternate statistical method that could have been used. The methods section should be detailed enough for reviewers to identify which analytical techniques were used (in particular, regression or stratification) and how specific confounders were measured.

For studies utilizing regression analysis, it is useful to identify if the study identified which variables were included and how they related to the outcome. If stratification was the analytical approach used, were the strata of analysis defined by the specified variables? Additionally, it is also important to assess the appropriateness of the analytical strategy in terms of the assumptions associated with the approach as differing methods of analysis are based on differing assumptions about the data and how it will respond.

© JBI, 2020. All rights reserved. JBI grants use of these tools for research purposes only. All other enquiries should be sent to jbisynthesis@adelaide.edu.au.

JBI CRITICAL APPRAISAL CHECKLIST FOR QUALITATIVE RESEARCH

Reviewer_______________________________________Date________________________________

Author____Year_________Record Number_________

	Yes	No	Unclear	Not applicable
1. Is there congruity between the stated philosophical perspective and the research methodology?	□	□	□	□
2. Is there congruity between the research methodology and the research question or objectives?	□	□	□	□
3. Is there congruity between the research methodology and the methods used to collect data?	□	□	□	□
4. Is there congruity between the research methodology and the representation and analysis of data?	□	□	□	□
5. Is there congruity between the research methodology and the interpretation of results?	□	□	□	□
6. Is there a statement locating the researcher culturally or theoretically?	□	□	□	□
7. Is the influence of the researcher on the research, and vice- versa, addressed?	□	□	□	□
8. Are participants, and their voices, adequately represented?	□	□	□	□
9. Is the research ethical according to current criteria or, for recent studies, and is there evidence of ethical approval by an appropriate body?	□	□	□	□
10. Do the conclusions drawn in the research report flow from the analysis, or interpretation, of the data?	□	□	□	□

Overall appraisal: Include □ Exclude □ Seek further info □

Comments (Including reason for exclusion)

© JBI, 2020. All rights reserved. JBI grants use of these tools for research purposes only. All other enquiries should be sent to jbisynthesis@adelaide.edu.au.

DISCUSSION OF CRITICAL APPRAISAL CRITERIA [106]

1. **Congruity between the stated philosophical perspective and the research methodology**

   Does the report clearly state the philosophical or theoretical premises on which the study is based? Does the report clearly state the methodological approach adopted on which the study is based? Is there congruence between the two? For example:

   A report may state that the study adopted a critical perspective and participatory action research methodology was followed. Here there is congruence between a critical view (focusing on knowledge arising out of critique, action and reflection) and action research (an approach that focuses on firstly working with groups to reflect on issues or practices, then considering how they could be different; then acting to create a change; and finally identifying new knowledge arising out of the action taken). However, a report may state that the study adopted an interpretive perspective and used survey methodology. Here there is incongruence between an interpretive view (focusing on knowledge arising out of studying what phenomena mean to individuals or groups) and surveys (an approach that focuses on asking standard questions to a defined study population); a report may state that the study was qualitative or used qualitative methodology (such statements do not demonstrate rigour in design) or make no statement on philosophical orientation or methodology.

2. **Congruity between the research methodology and the research question or objectives**

   Is the study methodology appropriate for addressing the research question? For example: A report may state that the research question was to seek understandings of the meaning of pain in a group of people with rheumatoid arthritis and that a phenomenological approach was taken. Here, there is congruity between this question and the methodology. A report may state that the research question was to establish the effects of counselling on the severity of pain experience and that an ethnographic approach was pursued. A question that tries to establish cause-and effect cannot be addressed by using an ethnographic approach (as ethnography sets out to develop understandings of cultural practices) and thus, this would be incongruent.

3. **Congruity between the research methodology and the methods used to collect data**

   Are the data collection methods appropriate to the methodology? For example:

   A report may state that the study pursued a phenomenological approach and data was collected through phenomenological interviews. There is congruence between the methodology and data collection; a report may state that the study pursued a phenomenological approach and data was collected through a postal questionnaire. There is incongruence between the methodology and data collection here as phenomenology seeks to elicit rich descriptions of the experience of a phenomena that cannot be achieved through seeking written responses to standardized questions.

4. **Congruity between the research methodology and the representation and analysis of data**

Are the data analyzed and represented in ways that are congruent with the stated methodological position? For example:

A report may state that the study pursued a phenomenological approach to explore people's experience of grief by asking participants to describe their experiences of grief. If the text generated from asking these questions is searched to establish the meaning of grief to participants, and the meanings of all participants are included in the report findings, then this represents congruity; the same report may, however, focus only on those meanings that were common to all participants and discard single reported meanings. This would not be appropriate in phenomenological work.

5. **There is congruence between the research methodology and the interpretation of results**

Are the results interpreted in ways that are appropriate to the methodology? For example:

A report may state that the study pursued a phenomenological approach to explore people's experience of facial disfigurement and the results are used to inform practitioners about accommodating individual differences in care. There is congruence between the methodology and this approach to interpretation; a report may state that the study pursued a phenomenological approach to explore people's experience of facial disfigurement and the results are used to generate practice checklists for assessment. There is incongruence between the methodology and this approach to interpretation as phenomenology seeks to understand the meaning of a phenomenon for the study participants and cannot be interpreted to suggest that this can be generalized to total populations to a degree where standardized assessments will have relevance across a population.

6. **Locating the researcher culturally or theoretically**

Are the beliefs and values, and their potential influence on the study declared? For example:

The researcher plays a substantial role in the qualitative research process and it is important, in appraising evidence that is generated in this way, to know the researcher's cultural and theoretical orientation. A high quality report will include a statement that clarifies this.

7. **Influence of the researcher on the research, and vice-versa, is addressed**

Is the potential for the researcher to influence the study and for the potential of the research process itself to influence the researcher and her/his interpretations acknowledged and addressed? For example:

Is the relationship between the researcher and the study participants addressed? Does the researcher critically examine her/his own role and potential influence during data collection? Is it reported how the researcher responded to events that arose during the study?

8. **Representation of participants and their voices**

   Generally, reports should provide illustrations from the data to show the basis of their conclusions and to ensure that participants are represented in the report.

9. **Ethical approval by an appropriate body**

   A statement on the ethical approval process followed should be in the report.

10. **Relationship of conclusions to analysis, or interpretation of the data**

    This criterion concerns the relationship between the findings reported and the views or words of study participants. In appraising a paper, appraisers seek to satisfy themselves that the conclusions drawn by the research are based on the data collected; data being the text generated through observation, interviews or other processes.

© JBI, 2020. All rights reserved. JBI grants use of these tools for research purposes only. All other enquiries should be sent to jbisynthesis@adelaide.edu.au.

JBI CRITICAL APPRAISAL CHECKLIST FOR STUDIES REPORTING PREVALENCE DATA

Reviewer______________________________________Date______________________________

Author_______________________________________Year_________ Record Number_________

	Yes	No	Unclear	Not applicable
1. Was the sample frame appropriate to address the target population?	□	□	□	□
2. Were study participants sampled in an appropriate way?	□	□	□	□
3. Was the sample size adequate?	□	□	□	□
4. Were the study subjects and the setting described in detail?	□	□	□	□
5. Was the data analysis conducted with sufficient coverage of the identified sample?	□	□	□	□
6. Were valid methods used for the identification of the condition?	□	□	□	□
7. Was the condition measured in a standard, reliable way for all participants?	□	□	□	□
8. Was there appropriate statistical analysis?	□	□	□	□
9. Was the response rate adequate, and if not, was the low response rate managed appropriately?	□	□	□	□

Overall appraisal: Include □ Exclude □ Seek further info □

Comments (Including reason for exclusion)

____

____

____

© JBI, 2020. All rights reserved. JBI grants use of these tools for research purposes only. All other enquiries should be sent to jbisynthesis@adelaide.edu.au.

JBI CRITICAL APPRAISAL CHECKLIST FOR STUDIES REPORTING PREVALENCE DATA [107]

Answers: Yes, No, Unclear or Not/Applicable

1. **Was the sample frame appropriate to address the target population?**

   This question relies upon knowledge of the broader characteristics of the population of interest and the geographical area. If the study is of women with breast cancer, knowledge of at least the characteristics, demographics and medical history is needed. The term "target population" should not be taken to infer every individual from everywhere or with similar disease or exposure characteristics. Instead, give consideration to specific population characteristics in the study, including age range, gender, morbidities, medications, and other potentially influential factors. For example, a sample frame may not be appropriate to address the target population if a certain group has been used (such as those working for one organisation, or one profession) and the results then inferred to the target population (i.e. working adults). A sample frame may be appropriate when it includes almost all the members of the target population (i.e. a census, or a complete list of participants or complete registry data).

2. **Were study participants recruited in an appropriate way?**

   Studies may report random sampling from a population, and the methods section should report how sampling was performed. Random probabilistic sampling from a defined subset of the population (sample frame) should be employed in most cases, however, random probabilistic sampling is not needed when everyone in the sampling frame will be included/ analysed. For example, reporting on all the data from a good census is appropriate as a good census will identify everybody. When using cluster sampling, such as a random sample of villages within a region, the methods need to be clearly stated as the precision of the final prevalence estimate incorporates the clustering effect. Convenience samples, such as a street survey or interviewing lots of people at a public gatherings are not considered to provide a representative sample of the base population.

3. **Was the sample size adequate?**

   The larger the sample, the narrower will be the confidence interval around the prevalence estimate, making the results more precise. An adequate sample size is important to ensure good precision of the final estimate. Ideally we are looking for evidence that the authors conducted a sample size calculation to determine an adequate sample size. This will estimate how many subjects are needed to produce a reliable estimate of the measure(s) of interest. For conditions with a low prevalence, a larger sample size is needed. Also consider sample sizes for subgroup (or characteristics) analyses, and whether these are appropriate. Sometimes, the study will be large enough (as in large national surveys) whereby a sample size calculation is not required. In these cases, sample size can be considered adequate.

When there is no sample size calculation and it is not a large national survey, the reviewers may consider conducting their own sample size analysis using the following formula: [108,109]

n= Z2P(1-P)

d2

Where:

- n= sample size
- Z = Z statistic for a level of confidence
- P = Expected prevalence or proportion (in proportion of one; if 20%, P = 0.2)
- d = precision (in proportion of one; if 5%, d=0.05)

4. Were the study subjects and setting described in detail?

Certain diseases or conditions vary in prevalence across different geographic regions and populations (e.g. Women vs. Men, sociodemographic variables between countries). The study sample should be described in sufficient detail so that other researchers can determine if it is comparable to the population of interest to them.

5. Was data analysis conducted with sufficient coverage of the identified sample?

Coverage bias can occur when not all subgroups of the identified sample respond at the same rate. For instance, you may have a very high response rate overall for your study, but the response rate for a certain subgroup (i.e. older adults) may be quite low.

6. Were valid methods used for the identification of the condition?

Here we are looking for measurement or classification bias. Many health problems are not easily diagnosed or defined and some measures may not be capable of including or excluding appropriate levels or stages of the health problem. If the outcomes were assessed based on existing definitions or diagnostic criteria, then the answer to this question is likely to be yes. If the outcomes were assessed using observer reported, or self-reported scales, the risk of over- or under-reporting is increased, and objectivity is compromised. Importantly, determine if the measurement tools used were validated instruments as this has a significant impact on outcome assessment validity.

7. Was the condition measured in a standard, reliable way for all participants?

Considerable judgment is required to determine the presence of some health outcomes. Having established the validity of the outcome measurement instrument (see item 6 of this scale), it is important to establish how the measurement was conducted. Were those involved in collecting data trained or educated in the use of the instrument/s? If there was more than one data collector, were they similar in terms of level of education, clinical or research experience, or level of responsibility in the piece of research being appraised? When there was more than one observer or collector, was there comparison of results from across the observers? Was the condition measured in the same way for all participants?

8. Was there appropriate statistical analysis?

Importantly, the numerator and denominator should be clearly reported, and percentages should be given with confidence intervals. The methods section should be detailed enough for reviewers to identify the analytical technique used and how specific variables were measured. Additionally, it is also important to assess the appropriateness of the analytical strategy in terms of the assumptions associated with the approach as differing methods of analysis are based on differing assumptions about the data and how it will respond.

9. Was the response rate adequate, and if not, was the low response rate managed appropriately?

A large number of dropouts, refusals or "not founds" amongst selected subjects may diminish a study's validity, as can a low response rates for survey studies. The authors should clearly discuss the response rate and any reasons for non-response and compare persons in the study to those not in the study, particularly with regards to their socio-demographic characteristics. If reasons for non-response appear to be unrelated to the outcome measured and the characteristics of non-responders are comparable to those who do respond in the study (addressed in question 5, coverage bias), the researchers may be able to justify a more modest response rate.

© JBI, 2020. All rights reserved. JBI grants use of these tools for research purposes only. All other enquiries should be sent to jbisynthesis@adelaide.edu.au.

JBI CRITICAL APPRAISAL CHECKLIST FOR COHORT STUDIES

Reviewer______________________________________Date______________________________

Author_______________________________________Year_________ Record Number_________

	Yes	No	Unclear	Not applicable
1. Were the two groups similar and recruited from the same population?	□	□	□	□
2. Were the exposures measured similarly to assign people to both exposed and unexposed groups?	□	□	□	□
3. Was the exposure measured in a valid and reliable way?	□	□	□	□
4. Were confounding factors identified?	□	□	□	□
5. Were strategies to deal with confounding factors stated?	□	□	□	□
6. Were the groups/participants free of the outcome at the start of the study (or at the moment of exposure)?	□	□	□	□
7. Were the outcomes measured in a valid and reliable way?	□	□	□	□
8. Was the follow up time reported and sufficient to be long enough for outcomes to occur?	□	□	□	□
9. Was follow up complete, and if not, were the reasons to loss to follow up described and explored?	□	□	□	□
10. Were strategies to address incomplete follow up utilized?	□	□	□	□
11. Was appropriate statistical analysis used?	□	□	□	□

Overall appraisal: Include □ Exclude □ Seek further info □

Comments (Including reason for exclusion)

____

____

© JBI, 2020. All rights reserved. JBI grants use of these tools for research purposes only. All other enquiries should be sent to jbisynthesis@adelaide.edu.au.

EXPLANATION OF COHORT STUDIES CRITICAL APPRAISAL [105]

Cohort Studies Critical Appraisal Tool

Answers: Yes, No, Unclear or Not/Applicable

1. Were the two groups similar and recruited from the same population?

Check the paper carefully for descriptions of participants to determine if patients within and across groups have similar characteristics in relation to exposure (e.g. risk factor under investigation). The two groups selected for comparison should be as similar as possible in all characteristics except for their exposure status, relevant to the study in question. The authors should provide clear inclusion and exclusion criteria that they developed prior to recruitment of the study participants.

2. Were the exposures measured similarly to assign people to both exposed and unexposed groups?

A high quality study at the level of cohort design should mention or describe how the exposures were measured. The exposure measures should be clearly defined and described in detail. This will enable reviewers to assess whether or not the participants received the exposure of interest.

3. Was the exposure measured in a valid and reliable way?

The study should clearly describe the method of measurement of exposure. Assessing validity requires that a 'gold standard' is available to which the measure can be compared. The validity of exposure measurement usually relates to whether a current measure is appropriate or whether a measure of past exposure is needed.

Reliability refers to the processes included in an epidemiological study to check repeatability of measurements of the exposures. These usually include intra-observer reliability and inter-observer reliability.

4. Were confounding factors identified?

Confounding has occurred where the estimated intervention exposure effect is biased by the presence of some difference between the comparison groups (apart from the exposure investigated/of interest). Typical confounders include baseline characteristics, prognostic factors, or concomitant exposures (e.g. smoking). A confounder is a difference between the comparison groups and it influences the direction of the study results. A high quality study at the level of cohort design will identify the potential confounders and measure them (where possible). This is difficult for studies where behavioral, attitudinal or lifestyle factors may impact on the results.

5. Were strategies to deal with confounding factors stated?

Strategies to deal with effects of confounding factors may be dealt within the study design or in data analysis. By matching or stratifying sampling of participants, effects of confounding factors can be adjusted for. When dealing with adjustment in data analysis, assess the statistics used in the study. Most will be some form of multivariate regression analysis to account for the confounding factors measured. Look out for a description of statistical methods as

regression methods such as logistic regression are usually employed to deal with confounding factors/variables of interest.

6. Were the groups/participants free of the outcome at the start of the study (or at the moment of exposure)?

The participants should be free of the outcomes of interest at the start of the study. Refer to the 'methods' section in the paper for this information, which is usually found in descriptions of participant/sample recruitment, definitions of variables, and/or inclusion/exclusion criteria.

7. Were the outcomes measured in a valid and reliable way?

Read the methods section of the paper. If for e.g. lung cancer is assessed based on existing definitions or diagnostic criteria, then the answer to this question is likely to be yes. If lung cancer is assessed using observer reported, or self-reported scales, the risk of over- or under-reporting is increased, and objectivity is compromised. Importantly, determine if the measurement tools used were validated instruments as this has a significant impact on outcome assessment validity.

Having established the objectivity of the outcome measurement (e.g. lung cancer) instrument, it's important to establish how the measurement was conducted. Were those involved in collecting data trained or educated in the use of the instrument/s? (e.g. radiographers). If there was more than one data collector, were they similar in terms of level of education, clinical or research experience, or level of responsibility in the piece of research being appraised?

8. Was the follow up time reported and sufficient to be long enough for outcomes to occur?

The appropriate length of time for follow up will vary with the nature and characteristics of the population of interest and/or the intervention, disease or exposure. To estimate an appropriate duration of follow up, read across multiple papers and take note of the range for duration of follow up. The opinions of experts in clinical practice or clinical research may also assist in determining an appropriate duration of follow up. For example, a longer timeframe may be needed to examine the association between occupational exposure to asbestos and the risk of lung cancer. It is important, particularly in cohort studies that follow up is long enough to enable the outcomes. However, it should be remembered that the research question and outcomes being examined would probably dictate the follow up time.

9. Was follow up complete, and if not, were the reasons to loss to follow up described and explored?

It is important in a cohort study that a greater percentage of people are followed up. As a general guideline, at least 80% of patients should be followed up. Generally a dropout rate of 5% or less is considered insignificant. A rate of 20% or greater is considered to significantly impact on the validity of the study. However, in observational studies conducted over a lengthy period of time a higher dropout rate is to be expected. A decision on whether to include or exclude a study because of a high dropout rate is a matter of judgement based on the reasons why people dropped out, and whether dropout rates were comparable in the exposed and unexposed groups.

Reporting of efforts to follow up participants that dropped out may be regarded as an indicator of a well conducted study. Look for clear and justifiable description of why people were left out, excluded, dropped out etc. If there is no clear description or a statement in this regards, this will be a 'No'.

10. Were strategies to address incomplete follow up utilized?

Some people may withdraw due to change in employment or some may die; however, it is important that their outcomes are assessed. Selection bias may occur as a result of incomplete follow up. Therefore, participants with unequal follow up periods must be taken into account in the analysis, which should be adjusted to allow for differences in length of follow up periods. This is usually done by calculating rates which use person-years at risk, i.e. considering time in the denominator.

11. Was appropriate statistical analysis used?

As with any consideration of statistical analysis, consideration should be given to whether there was a more appropriate alternate statistical method that could have been used. The methods section of cohort studies should be detailed enough for reviewers to identify which analytical techniques were used (in particular, regression or stratification) and how specific confounders were measured.

For studies utilizing regression analysis, it is useful to identify if the study identified which variables were included and how they related to the outcome. If stratification was the analytical approach used, were the strata of analysis defined by the specified variables? Additionally, it is also important to assess the appropriateness of the analytical strategy in terms of the assumptions associated with the approach as differing methods of analysis are based on differing assumptions about the data and how it will respond.

© JBI, 2020. All rights reserved. JBI grants use of these tools for research purposes only. All other enquiries should be sent to jbisynthesis@adelaide.edu.au.

JBI CRITICAL APPRAISAL CHECKLIST FOR SYSTEMATIC REVIEWS AND RESEARCH SYNTHESES

Reviewer______________________________________Date______________________________

Author_______________________________________Year_________ Record Number_________

	Yes	No	Unclear	Not applicable
1. Is the review question clearly and explicitly stated?	□	□	□	□
2. Were the inclusion criteria appropriate for the review question?	□	□	□	□
3. Was the search strategy appropriate?	□	□	□	□
4. Were the sources and resources used to search for studies adequate?	□	□	□	□
5. Were the criteria for appraising studies appropriate?	□	□	□	□
6. Was critical appraisal conducted by two or more reviewers independently?	□	□	□	□
7. Were there methods to minimize errors in data extraction?	□	□	□	□
8. Were the methods used to combine studies appropriate?	□	□	□	□
9. Was the likelihood of publication bias assessed?	□	□	□	□
10. Were recommendations for policy and/or practice supported by the reported data?	□	□	□	□
11. Were the specific directives for new research appropriate?	□	□	□	□

Overall appraisal: Include □ Exclude □ Seek further info □

Comments (Including reason for exclusion)

____

____

© JBI, 2020. All rights reserved. JBI grants use of these tools for research purposes only.
All other enquiries should be sent to jbisynthesis@adelaide.edu.au.

JBI CRITICAL APPRAISAL CHECKLIST FOR SYSTEMATIC REVIEWS AND RESEARCH SYNTHESES [110, 111]

When conducting an umbrella review using the JBI method, the critical appraisal instrument for Systematic Reviews should be used.

The primary and secondary reviewer should discuss each item in the appraisal instrument for each study included in their review. In particular, discussions should focus on what is considered acceptable to the aims of the review in terms of the specific study characteristics. When appraising systematic reviews this discussion may include issues such as what represents an adequate search strategy or appropriate methods of synthesis. The reviewers should be clear on what constitutes acceptable levels of information to allocate a positive appraisal compared with a negative, or response of “unclear”. This discussion should ideally take place before the reviewers independently conduct the appraisal.

Within umbrella reviews, quantitative or qualitative systematic reviews may be incorporated, as well as meta-analyses of existing research. There are 11 questions to guide the appraisal of systematic reviews or meta-analyses. Each question should be answered as “yes”, “no”, or “unclear”. Not applicable “NA” is also provided as an option and may be appropriate in rare instances.

1. **Is the review question clearly and explicitly stated?**

   The review question is an essential step in the systematic review process. A well-articulated question defines the scope of the review and aids in the development of the search strategy to locate the relevant evidence. An explicitly stated question, formulated around its PICO (Population, Intervention, Comparator, Outcome) elements aids both the review team in the conduct of the review and the reader in determining if the review has achieved its objectives. Ideally the review question should be articulated in a published protocol; however this will not always be the case with many reviews that are located.

2. **Were the inclusion criteria appropriate for the review question?**

   The inclusion criteria should be identifiable from, and match the review question. The necessary elements of the PICO should be explicit and clearly defined. The inclusion criteria should be detailed and the included reviews should clearly be eligible when matched against the stated inclusion criteria. Appraisers of meta-analyses will find that inclusion criteria may include criteria around the ability to conduct statistical analyses which would not be the norm for a systematic review. The types of included studies should be relevant to the review question, for example, an umbrella review aiming to summarize a range of effective non-pharmacological interventions for aggressive behaviors amongst elderly patients with dementia will limit itself to including systematic reviews and meta-analyses that synthesize quantitative studies assessing the various interventions; qualitative or economic reviews would not be included.

3. **Was the search strategy appropriate?**

   A systematic review should provide evidence of the search strategy that has been used to locate the evidence. This may be found in the methods section of the review report in some cases, or as an appendix that may be provided as supplementary information to the review publication. A systematic review should present a clear search strategy that addresses each of the identifiable PICO components of the review question. Some reviews may also provide a description of the approach to searching and how the terms that were ultimately used were derived, though due to limits on word counts in journals

this may be more the norm in online only publications. There should be evidence of logical and relevant keywords and terms and also evidence that Subject Headings and Indexing terms have been used in the conduct of the search. Limits on the search should also be considered and their potential impact; for example, if a date limit was used, was this appropriate and/or justified? If only English language studies were included, will such a language bias have an impact on the review? The response to these considerations will depend, in part, on the review question.

4. **Were the sources and resources used to search for studies adequate?**

   A systematic review should attempt to identify "all" the available evidence and as such there should be evidence of a comprehensive search strategy. Multiple electronic databases should be searched including major bibliographic citation databases such as MEDLINE and CINAHL. Ideally, other databases that are relevant to the review question should also be searched, for example, a systematic review with a question about a physical therapy intervention should also look to search the PEDro database, whilst a review focusing on an educational intervention should also search the ERIC. Reviews of effectiveness should aim to search trial registries. A comprehensive search is the ideal way to minimize publication bias, as a result, a well conducted systematic review should also attempt to search for grey literature, or "unpublished" studies; this may involve searching websites relevant to the review question, or thesis repositories.

5. **Were the criteria for appraising studies appropriate?**

   The systematic review should present a clear statement that critical appraisal was conducted and provide the details of the items that were used to assess the included studies. This may be presented in the methods of the review, as an appendix of supplementary information, or as a reference to a source that can be located. The tools or instruments used should be appropriate for the review question asked and the type of research conducted. For example, a systematic review of effectiveness should present a tool or instrument that addresses aspects of validity for experimental studies and randomized controlled trials such as randomization and blinding – if the review includes observational research to answer the same question a different tool would be more appropriate. Similarly, a review assessing diagnostic test accuracy may refer to the recognized QUADAS[1] tool.

6. **Was critical appraisal conducted by two or more reviewers independently?**

   Critical appraisal or some similar assessment of the quality of the literature included in a systematic review is essential. A key characteristic to minimize bias or systematic error in the conduct of a systematic review is to have the critical appraisal of the included studies completed independently and in duplicate by members of the review team. The systematic review should present a clear statement that critical appraisal was conducted by at least two reviewers working independently from each other and conferring where necessary to reach decision regarding study quality and eligibility on the basis of quality.

7. **Were there methods to minimize errors in data extraction?**

   Efforts made by review authors during data extraction can also minimize bias or systematic errors in the conduct of a systematic review. Strategies to minimize bias may include conducting all data extraction in duplicate and independently, using specific tools or instruments to guide data extraction and some evidence of piloting or training around their use.

8. **Were the methods used to combine studies appropriate?**

   A synthesis of the evidence is a key feature of a systematic review. The synthesis that is presented should be appropriate for the review question and the stated type of systematic review and evidence it refers to. If a meta-analysis has been conducted this needs to be reviewed carefully.

   Was it appropriate to combine the studies? Have the reviewers assessed heterogeneity statistically and provided some explanation for heterogeneity that may be present? Often, where heterogeneous studies are included in the systematic review, narrative synthesis will be an appropriate method for presenting the results of multiple studies. If a qualitative review, are the methods that have been used to synthesize findings congruent with the stated methodology of the review? Is there adequate descriptive and explanatory information to support the final synthesized findings that have been constructed from the findings sourced from the original research?

9. **Was the likelihood of publication bias assessed?**

   As mentioned, a comprehensive search strategy is the best means by which a review author may alleviate the impact of publication bias on the results of the review. Reviews may also present statistical tests such as Egger's test or funnel plots to also assess the potential presence of publication bias and its potential impact on the results of the review. This question will not be applicable to systematic reviews of qualitative evidence.

10. **Were recommendations for policy and/or practice supported by the reported data?**

    Whilst the first nine (9) questions specifically look to identify potential bias in the conduct of a systematic review, the final questions are more indictors of review quality rather than validity. Ideally a review should present recommendations for policy and practice. Where these recommendations are made there should be a clear link to the results of the review. Is there evidence that the strength of the findings and the quality of the research been considered in the formulation of review recommendations?

11. **Were the specific directives for new research appropriate?**

    The systematic review process is recognized for its ability to identify where gaps in the research, or knowledge base, around a particular topic exist. Most systematic review authors will provide some indication, often in the discussion section of the report, of where future research direction should lie. Where evidence is scarce or sample sizes that support overall estimates of effect are small and effect estimates are imprecise, repeating similar research to those identified by the review may be necessary and appropriate. In other instances, the case for new research questions to investigate the topic may be warranted.

© JBI, 2020. All rights reserved. JBI grants use of these tools for research purposes only. All other enquiries should be sent to jbisynthesis@adelaide.edu.au.

JBI CRITICAL APPRAISAL CHECKLIST FOR QUASI-EXPERIMENTAL STUDIES

RoB Assessor:	Date of Appraisal:	Record Number:
Study Author:	Study Title:	Study Year:

Internal Validity		Choice - Comments/Justification	Yes	No	Unclear	N/A
**Bias related to temporal precedence**						
1	Is it clear in the study what is the "cause" and what is the "effect" (i.e. there is no confusion about which variable comes first)?		☐	☐	☐	☐
**Bias related to selection and allocation**						
2	Was there a control group?		☐	☐	☐	☐
**Bias related to confounding factors**						
3	Were participants included in any comparisons similar?		☐	☐	☐	☐
**Bias related to administration of intervention/exposure**						

4	**Were the participants included in any comparisons receiving similar treatment/care, other than the exposure or intervention of interest?**		☐	☐	☐	☐

**Bias related to assessment, detection and measurement of the outcome**						
5	**Were there multiple measurements of the outcome, both pre and post the intervention/exposure?**		**Yes**	**No**	**Unclear**	**N/A**
	**Outcome 1**		☐	☐	☐	☐
	**Outcome 2**		☐	☐	☐	☐
	**Outcome 3**		☐	☐	☐	☐
	**Outcome 4**		☐	☐	☐	☐
	**Outcome 5**		☐	☐	☐	☐
	**Outcome 6**		☐	☐	☐	☐
	**Outcome 7**		☐	☐	☐	☐

6	**Were the outcomes of participants included in any comparisons measured in the same way?**		**Yes**	**No**	**Unclear**	**N/A**
	**Outcome 1**		☐	☐	☐	☐
	**Outcome 2**		☐	☐	☐	☐

	**Outcome 3**		☐	☐	☐	☐
	**Outcome 4**		☐	☐	☐	☐
	**Outcome 5**		☐	☐	☐	☐
	**Outcome 6**		☐	☐	☐	☐
	**Outcome 7**		☐	☐	☐	☐

7	**Were outcomes measured in a reliable way?**		**Yes**	**No**	**Unclear**	**N/A**
	**Outcome 1**		☐	☐	☐	☐
	**Outcome 2**		☐	☐	☐	☐
	**Outcome 3**		☐	☐	☐	☐
	**Outcome 4**		☐	☐	☐	☐
	**Outcome 5**		☐	☐	☐	☐
	**Outcome 6**		☐	☐	☐	☐
	**Outcome 7**		☐	☐	☐	☐

Bias related to participant retention

8	**Was follow-up complete and if not, were differences between groups in terms of their follow-up adequately described and analyzed?**					
	**Outcome 1**		**Yes**	**No**	**Unclear**	**N/A**
	Result 1		☐	☐	☐	☐
	Result 2		☐	☐	☐	☐
	Result 3		☐	☐	☐	☐
	**Outcome 2**		**Yes**	**No**	**Unclear**	**N/A**
	Result 1		☐	☐	☐	☐
	Result 2		☐	☐	☐	☐
	Result 3		☐	☐	☐	☐
	**Outcome 3**		**Yes**	**No**	**Unclear**	**N/A**
	Result 1		☐	☐	☐	☐
	Result 2		☐	☐	☐	☐
	Result 3		☐	☐	☐	☐
	**Outcome 4**		**Yes**	**No**	**Unclear**	**N/A**
	Result 1		☐	☐	☐	☐
	Result 2		☐	☐	☐	☐

Result 3		☐	☐	☐	☐
**Outcome 5**		**Yes**	**No**	**Unclear**	**N/A**
Result 1		☐	☐	☐	☐
Result 2		☐	☐	☐	☐
Result 3		☐	☐	☐	☐
**Outcome 6**		**Yes**	**No**	**Unclear**	**N/A**
Result 1		☐	☐	☐	☐
Result 2		☐	☐	☐	☐
Result 3		☐	☐	☐	☐
**Outcome 7**		**Yes**	**No**	**Unclear**	**N/A**
Result 1		☐	☐	☐	☐
Result 2		☐	☐	☐	☐
Result 3		☐	☐	☐	☐

Statistical Conclusion Validity

9	**Was appropriate statistical analysis used?**					
	**Outcome 1**		**Yes**	**No**	**Unclear**	**N/A**

Result 1		☐	☐	☐	☐
Result 2		☐	☐	☐	☐
Result 3		☐	☐	☐	☐
**Outcome 2**		**Yes**	**No**	**Unclear**	**N/A**
Result 1		☐	☐	☐	☐
Result 2		☐	☐	☐	☐
Result 3		☐	☐	☐	☐
**Outcome 3**		**Yes**	**No**	**Unclear**	**N/A**
Result 1		☐	☐	☐	☐
Result 2		☐	☐	☐	☐
Result 3		☐	☐	☐	☐
**Outcome 4**		**Yes**	**No**	**Unclear**	**N/A**
Result 1		☐	☐	☐	☐
Result 2		☐	☐	☐	☐
Result 3		☐	☐	☐	☐
**Outcome 5**		**Yes**	**No**	**Unclear**	**N/A**
Result 1		☐	☐	☐	☐

Result 2		☐	☐	☐	☐
Result 3		☐	☐	☐	☐
**Outcome 6**		**Yes**	**No**	**Unclear**	**N/A**
Result 1		☐	☐	☐	☐
Result 2		☐	☐	☐	☐
Result 3		☐	☐	☐	☐
**Outcome 7**		**Yes**	**No**	**Unclear**	**N/A**
Result 1		☐	☐	☐	☐
Result 2		☐	☐	☐	☐
Result 3		☐	☐	☐	☐

Overall appraisal: **Include:** ☐ **Exclude:** ☐ **Seek Further Info:** ☐

Comments:

© JBI, 2022. All rights reserved. JBI grants use of these tools for research purposes only. All other enquiries should be sent to jbisynthesis@adelaide.edu.au

Supplementary Material S2. Summary of Methodological Quality Assessment of Included Studies According to Study Design and Appraisal Tool (JBI or SANRA)

	First Author (Year)	Study Design	Tool Used	Items Met (%)	Methodological Quality	Key Observations
1	**Dobbs et al. (2020)**	Mixed Methods (Descriptive + Qualitative)	JBI Checklist for Analytical Cross-Sectional Studies (8 items) and JBI Checklist for Qualitative Research (10 items)	6/8 (75%) / 8/10 (80%)	Moderate to High Quality	Confounding factors not identified or controlled; lacks reflexivity regarding researcher influence.
2	**Fernández-García et al. (2020)**	Descriptive Cross-Sectional Study	JBI Checklist for Analytical Cross-Sectional Studies (8 items)	6/8 (75%)	Moderate to High Quality	Confounding factors were neither identified nor controlled.
3	**Huey et al. (2020)**	Narrative Review	SANDRA (12 items)	11/12 (87.5%)	Moderate to High Quality	Appropriate synthesis and scientific reasoning; more expository than analytical or critical narrative.
4	**Jun & Kim (2020)**	Descriptive Cross-Sectional Study	JBI Checklist for Analytical Cross-Sectional Studies (8 items)	8/8 (100%)	Excellent	No methodological concerns identified

5	**Natto (2020)**	Descriptive Cross-Sectional Study	JBI Checklist for Analytical Cross-Sectional Studies (8 items)	6/8 (75%)	Moderate to High Quality	Potential confounding factors were neither identified nor controlled.
6	**Prigitano et al. (2020)**	Descriptive Cross-Sectional Study	JBI Checklist for Analytical Cross-Sectional Studies (8 items)	6/8 (75%)	Moderate to High Quality	Potential confounding factors were neither identified nor controlled.
7	**Pulvers et al. (2020)**	Descriptive Cross-Sectional Study	JBI Checklist for Analytical Cross-Sectional Studies (8 items)	8/8 (100%)	Excellent	No methodological concerns identified
8	**Wamamili et al. (2020)**	Prevalence Study	JBI Checklist for Prevalence Studies (9 items)	7/8 (87.5%)	Moderate to High Quality	No strategies were implemented to control potential confounding factors.
9	**Wang et al. (2020)**	Descriptive Cross-Sectional Study	JBI Checklist for Analytical Cross-Sectional Studies (8 items)	8/8 (100%)	Excellent	No methodological concerns identified
10	**Almeida-da-Silva et al. (2021)**	Narrative Review	SANDRA (12 items)	11/12 (92%)	Moderate to High Quality	No information provided to ensure reproducibility.
11	**Alzahrani et al. (2021)**	Descriptive Cross-Sectional Study	JBI Checklist for Analytical Cross-Sectional Studies (8 items)	8/8 (100%)	Excellent	No methodological concerns identified

12	**Ganson & Nagata (2021)**	Descriptive Cross-Sectional Study	JBI Checklist for Analytical Cross-Sectional Studies (8 items)	8/8 (100%)	Excellent	No methodological concerns identified
13	**Jones et al. (2021)**	Descriptive Cross-Sectional Study	JBI Checklist for Analytical Cross-Sectional Studies (8 items)	6/8 (75%)	Moderate to High Quality	No confounding factors were controlled.
14	**Kurdi et al. (2021)**	Descriptive Cross-Sectional Study	JBI Checklist for Analytical Cross-Sectional Studies (8 items)	8/8 (100%)	Excellent	No methodological concerns identified
15	**Newcombe (2021)**	Descriptive Cross-Sectional Study	JBI Checklist for Analytical Cross-Sectional Studies (8 items)	6/8 (75%)	Moderate to High Quality	Potential confounding factors were neither identified nor controlled.
16	**Omoike & Johnson (2021)**	Descriptive Cross-Sectional Study	JBI Checklist for Analytical Cross-Sectional Studies (8 items)	8/8 (100%)	Excellent	No methodological concerns identified
17	**Oh et al. (2021)**	Descriptive Cross-Sectional Study	JBI Checklist for Analytical Cross-Sectional Studies (8 items)	8/8 (100%)	Excellent	No methodological concerns identified
18	**Páez et al. (2021)**	Descriptive Cross-Sectional Study	JBI Checklist for Analytical Cross-Sectional Studies (8 items)	6/8 (75%)	Moderate to High Quality	Absence of multivariate adjustment limits control over potential confounding factors.

19	**Phetphum et al. (2021)**	Descriptive Cross-Sectional Study	JBI Checklist for Analytical Cross-Sectional Studies (8 items)	8/8 (100%)	Excellent	No methodological concerns identified
20	**Pougnet et al. (2021)**	Descriptive Cross-Sectional Study	JBI Checklist for Analytical Cross-Sectional Studies (8 items)	5/8 (62.5%)	Moderate	The use of a validated questionnaire is not reported, nor is any form of validation of the instrument described. Potential confounding factors were neither identified nor controlled.
21	**Rayman & Kessler (2021)**	Mixed Methodological Study (Descriptive Cross-Sectional Study and Qualitative study).	JBI Checklist for Analytical Cross-Sectional Studies (8 items) JBI Checklist for Qualitative Research (10 items)	6/8 (75%) / 7/10 (70%)	Moderate to High Quality	The study lacks an explicit philosophical or theoretical framework and does not address reflexivity or the influence of the researchers on the research process.
22	**Tarran et al. (2021)**	Narrative Review	SANDRA (12 items)	11/12 (92%)	Moderate to High Quality	No clear information on how reproducibility was ensured.
23	**Worthen & Ahmad (2021)**	Descriptive Cross-Sectional Study	JBI Checklist for Analytical Cross-Sectional Studies (8 items)	7/8 (87.5%)	Moderate to High Quality	Confounding factors were acknowledged and examined through stratified analysis, but no multivariable adjustment was performed.
24	**AlMuhaissen et al. (2022)**	Descriptive Cross-Sectional Study	JBI Checklist for Analytical Cross-Sectional Studies (8 items)	8/8 (100%)	Excellent	No methodological concerns identified

25	**Babjaková et al. (2022)**	Descriptive Cross-Sectional Study	JBI Checklist for Analytical Cross-Sectional Studies (8 items)	8/8 (100%)	Excellent	No methodological concerns identified
26	**McLeish et al. (2022)**	Descriptive Cross-Sectional Study	JBI Checklist for Analytical Cross-Sectional Studies (8 items)	6/8 (75%)	Moderate to High Quality	Confounding factors were neither identified nor controlled.
27	**Seidel et al. (2022)**	Longitudinal Cohort Study	JBI Checklist for Cohort Studies (11 items)	11/11 (100%)	Excellent	No methodological concerns identified
28	**Hair et al. (2023)**	Descriptive Cross-Sectional Study	JBI Checklist for Analytical Cross-Sectional Studies (8 items)	8/8 (100%)	Excellent	No methodological concerns identified
29	**Holden & Simerson (2023)**	Quasi-experimental study	JBI Checklist for Quasi-Experimental Studies (9 items)	7/9 (77.8%)	Moderate to High Quality	The study lacked a control group, and the measurement tool used was not psychometrically validated.
30	**Kaewsutha & Karawekpanyawong (2023)**	Descriptive Cross-Sectional Study	JBI Checklist for Analytical Cross-Sectional Studies (8 items)	7/8 (87.5%)	Moderate to High Quality	Confounding factors were identified but not controlled.
31	**Resano et al. (2023)**	Descriptive Cross-Sectional Study	JBI Checklist for Analytical Cross-Sectional Studies (8 items)	7.5/8 (94%)	Moderate to High Quality	Adapted items from ITC-PATH, but no formal psychometric properties reported.

32	**Vilcassim et al. (2023)**	Descriptive Cross-Sectional Study	JBI Checklist for Analytical Cross-Sectional Studies (8 items)	6/8 (75%)	Moderate to High Quality	Ad hoc questionnaire used without formal validation.
33	**Albadrani et al. (2024)**	Systematic Review	JBI Checklist for Systematic Reviews (11 items)	11/11 (100%)	Excellent	No methodological concerns identified
34	**Bataineh et al. (2024)**	Descriptive Cross-Sectional Study	JBI Checklist for Analytical Cohort Studies (8 items)	8/8 (100%)	Excellent	No methodological concerns identified
35	**Folivi et al. (2024)**	Descriptive Cross-Sectional Study	JBI Checklist for Analytical Cross-Sectional Studies (8 items)	8/8 (100%)	Excellent	No methodological concerns identified
36	**Kinouani et al. (2024)**	Mixed Methodological Study (Descriptive Cross-Sectional Study and Qualitative study).	JBI Checklist for Analytical Cross-Sectional Studies (8 items) and JBI Checklist for Qualitative Research (10 items)	8/8 (100%) / 8/10 (80%)	Excellent / Moderate to High Quality	No methodological concerns identified / Lack of reflexivity regarding the researcher's role
37	**Maqsood et al. (2024)**	Descriptive Cross-Sectional Study	JBI Checklist for Analytical Cross-Sectional Studies (8 items)	8/8 (100%)	Excellent	No methodological concerns identified

38	**Mostafa & Taha (2024)**	Descriptive Cross-Sectional Study	JBI Checklist for Analytical Cross-Sectional Studies (8 items)	8/8 (100%)	Excellent	No methodological concerns identified
39	**Roh (2024)**	Descriptive Cross-Sectional Study	JBI Checklist for Analytical Cross-Sectional Studies (8 items)	8/8 (100%)	Excellent	No methodological concerns identified
40	**Singer et al. (2024)**	Longitudinal Cohort Study	JBI Checklist for Cohort Studies (11 items)	10/11 (91%)	Moderate to High Quality	Follow-up rate is not clearly reported.
41	**Ou et al. (2024)**	Prospective Cohort Study	JBI Checklist for Cohort Studies (11 items)	11/11 (100%)	Excellent	No methodological concerns identified
42	**Kajan et al. (2025)**	Descriptive Cross-Sectional Study	JBI Checklist for Analytical Cross-Sectional Studies (8 items)	8/8 (100%)	Excellent	No methodological concerns identified
43	**Soerianto & Jaspers (2025)**	Narrative Review	SANDRA (12 items)	12/12 (100%)	Excellent	No methodological concerns identified

JBI: Joanna Briggs Institute
SANRA: Scale for the Assessment of Narrative Review Articles.
